# Microwave‐Activated Bacterial Biorobot for Multimodal Cancer Therapy

**DOI:** 10.1002/advs.202504603

**Published:** 2025-08-11

**Authors:** Huilan Zhuang, Yongjian Zhang, Yajuan Fu, Dangjin Ke, Qi Chen, Sijie Shao, Panpan Xue, Yuanchun Chen, Xuemei Zeng, Shuangqian Yan

**Affiliations:** ^1^ Fujian Provincial Key Laboratory of Flexible Electronics Strait Institute of Flexible Electronics (SIFE Future Technologies) Fujian Normal University Fuzhou 350007 P. R. China; ^2^ The Sixth Affiliated Hospital of Harbin Medical University Harbin Heilongjiang 150000 China; ^3^ Key Laboratory of Innate Immune Biology of Fujian Province Biomedical Research Center of South China College of Life Sciences Fujian Normal University 1 Keji Road Fuzhou 350117 P. R. China

**Keywords:** bacteria therapy, cuproptosis, ferroptosis, immunotherapy, synthetic biology

## Abstract

Advances in synthetic biology have enabled innovative strategies for cancer therapy, yet precise control of therapeutic expression and biosafety remain critical challenges. To address these issues, a bacterial hybrid biorobot is developed using *Escherichia coli* (MG1655) engineered for localized activation by microwaves. Upon activation, the biorobot expresses glucose oxidase (GOx) at tumor sites, leading to glucose depletion and hydrogen peroxide generation. Surface‐attached Cu_2_O nanoparticles catalyze this hydrogen peroxide through a Fenton‐like reaction, producing reactive oxygen species that drive multiple forms of tumor cell death, including apoptosis, ferroptosis, and cuproptosis. Comprehensive in vitro and in vivo studies confirm the efficacy of this approach, while transcriptomic analysis reveals disruption of glucose metabolism and robust activation of antitumor immune responses. This work demonstrates the potential of this engineered bacterial platform as a safe and versatile tool for precise, multimodal cancer treatment.

## Graphical abstract

An *Escherichia coli* bacterial biorobot is engineered for localized microwave activation in cancer therapy. Upon activation at tumor sites, the biorobot releases glucose oxidase, inducing tumor cell death via glucose depletion and H_2_O_2_ generation catalyzed by Cu_2_O. In vitro and in vivo studies demonstrate disrupted glucose metabolism and potent antitumor immune responses, establishing a versatile and safe cancer treatment tool.

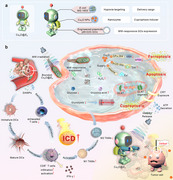

## 1 Introduction

Cancer remains a major global health challenge, with increasing incidence rates driven by aging populations and lifestyle factors.^[^ ^1^ ^]^ While conventional treatments such as radiation, chemotherapy, and surgery have shown effectiveness in specific contexts, they are often associated with significant side effects, limited efficacy against certain cancer types, and the emergence of drug resistance.^[^ ^2^ ^]^ This underscores an urgent need for innovative therapeutic strategies, particularly personalized approaches that leverage biotechnological advances such immunotherapy and targeted therapies.^[^ ^3^ ^]^ These emerging modalities aim to enhance treatment efficacy, minimize adverse effects, and provide comprehensive, patient‐specific options, offering the potential for transformative breakthroughs in cancer care.

A hallmark of cancer is the distinct metabolic reprograming of tumor cells, including dysregulation in glucose, lipid, and protein metabolism, as well as microenvironmental acidification and oxidative stress.^[^ ^4^ ^]^ These metabolic abnormalities influence tumor proliferation, invasion, and response to therapy, with aberrant glucose metabolism‐commonly known as the Warburg effect‐playing a pivotal role.^[^ ^5^ ^]^ This phenomenon refers to the preference of cancer cells for glycolysis over oxidative phosphorylation, even in the presence of oxygen, leading to increased glucose uptake and lactate production.^[^ ^6^ ^]^ Exploiting these metabolic vulnerabilities has become a critical focus in cancer therapy. Current strategies targeting tumor glucose metabolism include: 1) glycolysis inhibitors to disrupt cancer cell energy production, 2) inhibitors of glucose uptake, and 3) starvation‐based therapies.^[^ ^7^ ^]^ One promising approach involves the delivery of glucose oxidase (GOx) into tumors, where it catalyzes glucose conversion into gluconic acid and hydrogen peroxide (H_2_O_2_), selectively inducing oxidative stress in cancer cells.^[^ ^8^ ^]^ However, challenges such as delivery efficiency, immune responses, specificity, and adaptive resistance must be addressed to fully realize the therapeutic potential of GOx‐based interventions.^[^ ^9^ ^]^ Advances in research and technology are essential to overcome these barriers and optimize metabolic‐targeted therapies in oncology.

Bacteria, with their inherent tumor‐targeting capabilities and immunostimulatory properties, have emerged as a promising platform for therapeutic delivery.^[^ ^10^ ^]^ Advances in synthetic biology and gene editing have enabled the engineering of bacteria with precise functional modifications, allowing them to deliver drugs, activate therapeutics under specific conditions, or produce tumor‐targeting agents.^[^ ^11^ ^]^ Such approaches minimize damage to healthy tissues through targeted action.^[^ ^12^ ^]^ However, achieving precise control over in vivo gene expression is crucial for the safety and efficacy of bacterial therapies. Strategies for stimulus‐responsive gene expression in genetically modified bacteria include chemical,^[^ ^13^ ^]^ temperature,^[^ ^14^ ^]^ light,^[^ ^15^ ^]^ biological (e.g., quorum sensing),^[^ ^16^ ^]^ and ultrasound‐induced systems.^[^ ^17^ ^]^ Microwave (MW) thermotherapy, which uses MW energy to generate heat for localized treatment, has garnered significant attention due to its ability to penetrate tissues and selectively heat specific areas.^[^ ^18^ ^]^ This technology serves as an exogenous stimulus for temperature‐sensitive promoters, making it a valuable tool for bacterial therapies.

**Scheme 1:**
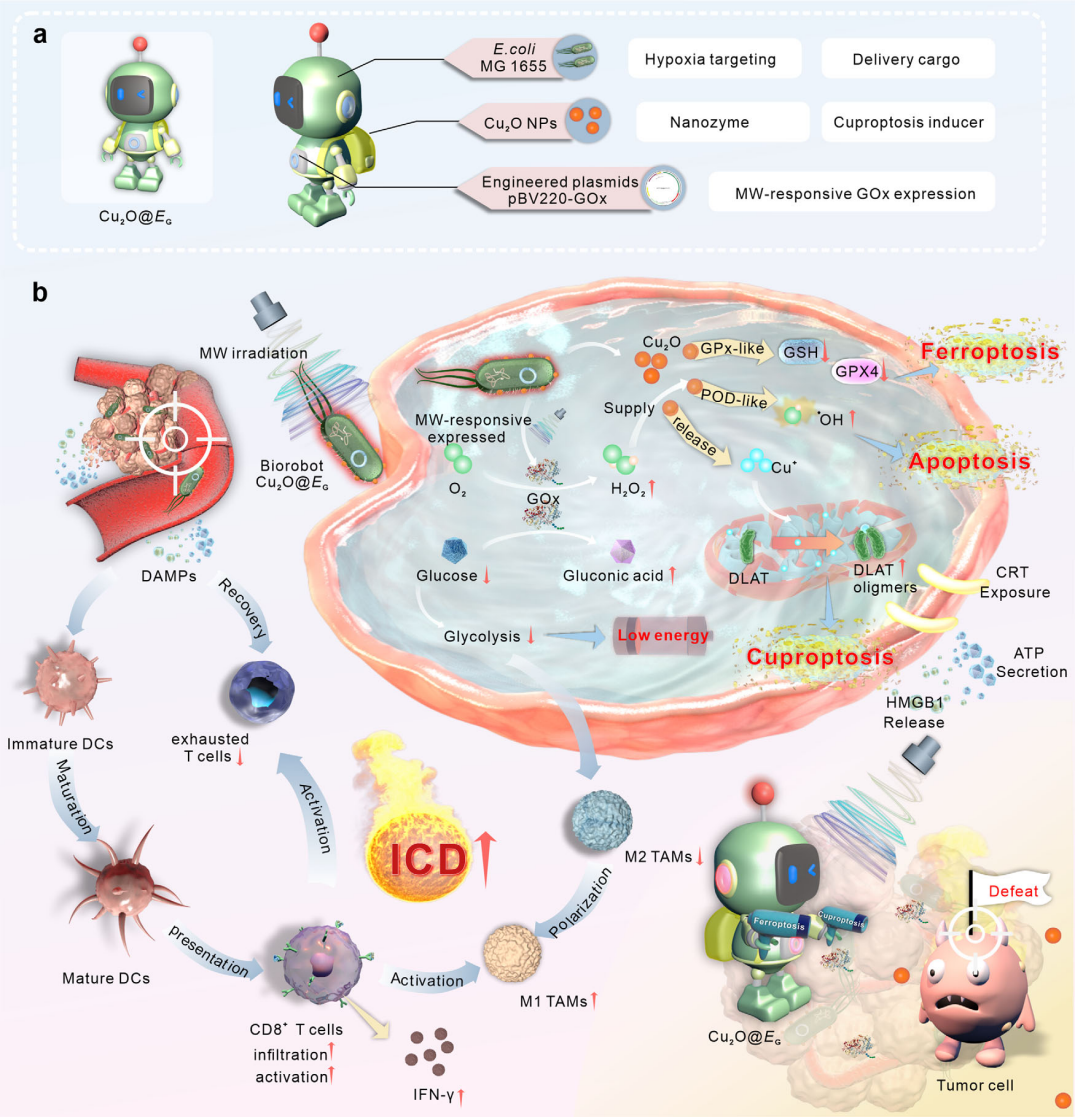
Schematic illustration. a) Overview of the key components of the biorobot Cu_2_O@*E*_G_. b) Schematic diagram of biorobot in controlling GOx expression by microwave and their mechanisms for cancer therapy.

In this study, we report the design of a biorobot, termed Cu_2_O@*E*_G_, for spatiotemporally controlled tumor therapy. The Cu_2_O@*E*_G_ biorobot combines a genetically engineered avirulent strain of *Escherichia coli* MG1655 (*E. coli*) with copper(I) oxide (Cu_2_O) nanoparticles (**Scheme** **1**a). This system integrates *E. coli* engineered for hypoxic tumor targeting and drug delivery with a microwave‐activated mechanism for GOx expression. Cu_2_O NPs, bound to the bacterial surface, exhibit nanozyme catalytic activity and induce cuproptosis.^[^ ^19^ ^]^ Upon MW stimulation, the biorobot activates a thermal switch, inducing GOx expression, which metabolizes glucose into gluconic acid H_2_O_2_. The generated H_2_O_2_ acts as a substrate for Cu_2_O NPs to produce reactive oxygen species (ROS) via a Fenton‐like reaction, exacerbating oxidative stress and intracellular redox imbalance.^[^ ^20^ ^]^ This cascade promotes apoptosis and ferroptosis in cancer cells. Additionally, Cu_2_O NPs directly trigger cuproptosis by facilitating the aggregation of lipoylated dihydrolipoamide S‐acetyltransferase (DLAT). In a breast tumor mouse model, Cu_2_O@*E*_G_ induced robust antitumor immune responses (Scheme 1b). Collectively, this biorobot achieves a multi‐therapeutic effect within a single treatment regimen, offering enhanced therapeutic efficacy. Our findings underscore the transformative potential of this bioengineered bacterial hybrid system, heralding a new frontier in cancer therapy.

## 2 Results

### 2.1 Composition and Characterization of biorobot

**Figure 1:**
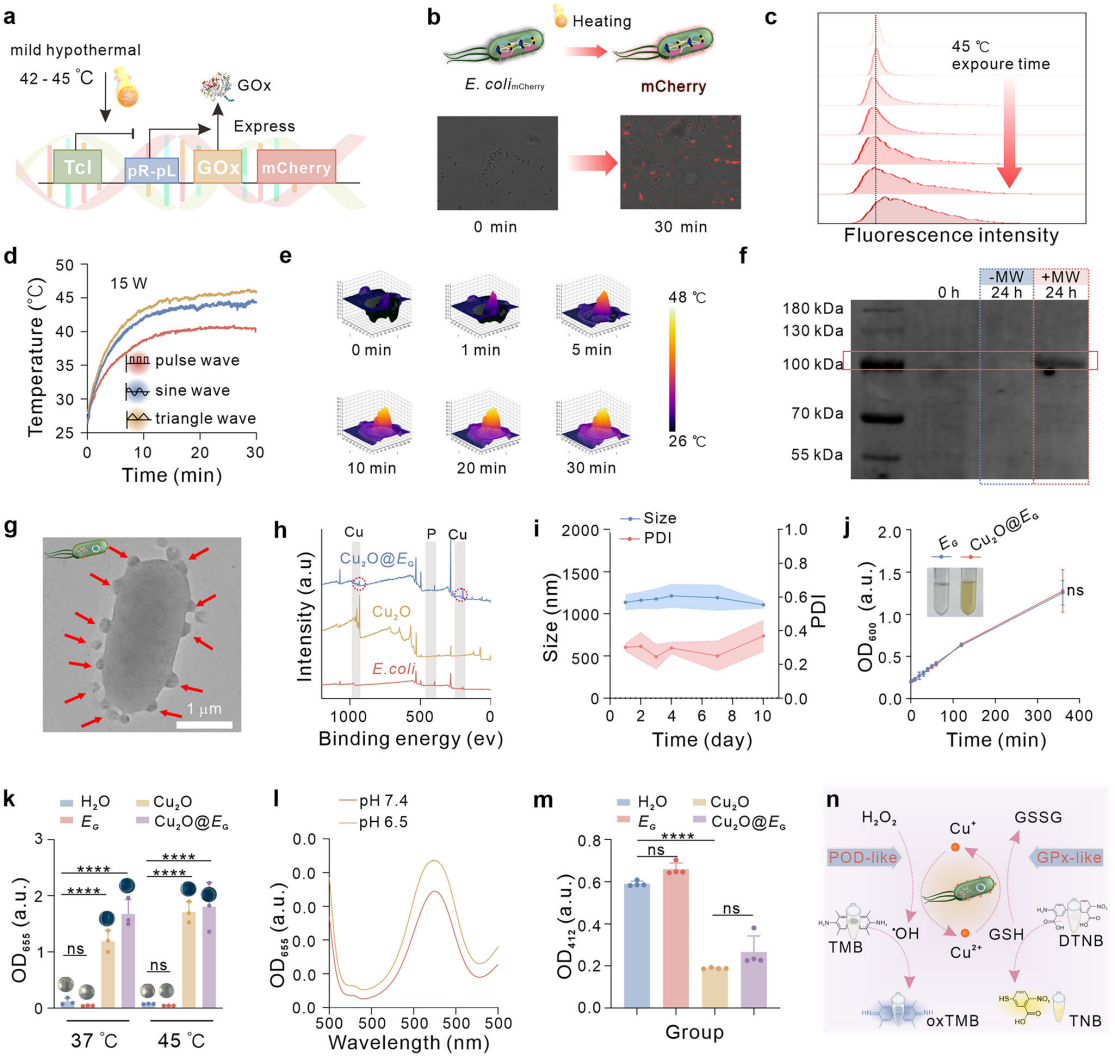
Contraction and characterization of Cu_2_O@*E*_G_. a) Schematic illustration of temperature‐responsive state switch modified to express GOx of the designed plasmid. b) Depiction of the genetic circuit utilized to evaluate the performance of temperature‐sensitive repressors in modified bacteria, accompanied by fluorescence images of mCherry protein expression in engineered bacteria at 45 °C for 0 and 30 min. c) Quantification of mCherry expression levels in engineered bacteria at 37 °C or 45 °C for varying time intervals using FCM. d) Temperature response of PBS solution subjected to microwave (4.95 GHz, 15 W) irradiation under different waveform conditions. All experiments were conducted independently three times, yielding consistent results. e) 3D infrared imaging of PBS solution following microwave treatment (triangle wave, 4.95 GHz, 15 W) at designated time intervals (0–30 min). f) Expression of GOx‐His tag protein by *E*_G_ under different treatments. g) TEM image of bacteria modified with Cu_2_O (Red arrows indicate Cu_2_O nanoparticles electrostatically adsorbed onto the surface of *E*_G_). h) XPS survey of *E. coli*, Cu_2_O, and Cu_2_O@*E*_G_. i) Changes in hydrodynamic diameter of Cu_2_O@*E*_G_ in PBS solution over a 10‐day period (*n* = 3). j) Growth characteristics of *E*_G_ and Cu_2_O@*E*_G_ in LB medium, with optical photographs illustrating *E*_G_ with and without Cu_2_O at the initial time point (0 min). (*n* = 3). k) ^•^OH generation of detected by TMB (*n* = 3). l) Measurement of ^•^OH generation by Cu_2_O@*E*_G_ under varying pH conditions. m) GSH consumption quantified using DTNB (*n* = 3). n) Schematic illustration of the dual peroxidase POD‐like and GPx‐like catalytic activity of Cu_2_O@*E*_G_. Data were presented as mean ± S.D. Statistical analysis was calculated by using one‐way analysis of variance with a Tukey's test (****p < 0.0001 and ns > 0.05).

To develop a precise and temporally controlled engineered bacterium, we first constructed plasmids based on the pBV220 plasmids, which includes a thermally sensitive Tcl promoter (Figure S1a, Supporting Information). The sequences encoding GOx and mCherry fluorescent reporter were placed downstream of the pR‐pL tandem promoter, enabling gene expression induction under mild heat stress (42–45 °C, **Figure** **1**a). These plasmids were then introduced into tumor‐targeting *E*. *coli* to generate the genetically engineered bacteria (designated as *E*_G_) (Figure S1b, Supporting Information). The inclusion of the mCherry fluorescent reporter gene facilitated real‐time visualization of gene circuit activation. Following a 30‐min incubation at 45 °C, bright red fluorescence was observed, confirming the successful construction and activation of the gene circuit (Figure 1b; Figure S2, Supporting Information). In contrast, negligible fluorescence was detected at 37 °C, demonstrating effective suppression of expression at this temperature. Prolonged incubation at 45 °C led to increased fluorescence intensity, indicating stable and sustained mCherry expression due to the relief of Tcl repression (Figure 1c).

To enable noninvasive and acute remotely controlled expression in the engineered bacteria, we leveraged the thermal effects of MW irradiation to locally activate the gene circuit. MW parameters, including waveform, power, and irradiation duration, were systematically optimized (Figure S3, Supporting Information). A triangle waveform at 15 W was identified as optimal, rapidly heating the solution to 45 °C within 5 min and maintaining this temperature during irradiation (Figures 1d,e). Subsequently, we investigated biosynthesis in *E*_G_ triggered by MW stimulation. The expression levels of GOx in bacterial supernatants were analysed via SDS‐PAGE. Distinct bands corresponding to his‐tagged GOx (100 kDa) were observed after 30 min of MW irradiation, compared to negligible expression in control groups without heat (37 °C) or MW stimulation (Figure 1f). Furthermore, bacterial viability remained unaffected following 30 min of treatment at 45 °C or MW induction (Figure S4, Supporting Information). These findings demonstrate the capacity of *E*_G_ to tightly regulate gene expression and secrete functional GOx protein under MW activation, highlighting its potential for therapeutic applications, including antitumor strategies.

Due to the limited efficacy of single therapeutic modalities, combining multiple approaches is often necessary for enhanced treatment outcomes. To leverage the ability GOx to generate H_2_O_2_ during glucose catalysis, we coated the Cu_2_O nanoparticles onto the surface of *E*_G_ to enable a cascade catalytic reaction that converts H_2_O_2_ into lethal ^•^OH for chemodynamical therapy.

Cu_2_O nanoparticles were synthesized following established protocols,^[^ ^19^ ^]^ and their successful formation of spherical particles with an average size of 50 nm was confirmed through X‐ray diffraction (XRD) and transmission electron microscopy (TEM) (Figure S5, Supporting Information). X‐ray photoelectron spectroscopy (XPS) analysis further revealed that most copper ions in Cu_2_O nanoparticles were monovalent (Figure S6, Supporting Information). After coupling Cu_2_O nanoparticles to *E*_G_ via electrostatic adsorption, their physicochemical properties were thoroughly characterized. TEM imaging demonstrated a uniform distribution of Cu_2_O nanoparticles on the surface of *E*_G_ (Figure 1g; Figure S7, Supporting Information), corroborated by dynamic light scattering (DLS) measurement, which showed an increase in the hydrated size of from *E*_G_ (851 nm) to 1064 nm (Cu_2_O@*E*_G_) (Figure S8, Supporting Information). Zeta potential analysis revealed a shift from −23.5 to −15.3 mV, indicating successful surface modification (Figure S9, Supporting Information). This was further supported by the appearance of copper‐specific peaks in the XPS spectrum of Cu_2_O@*E*_G_ (Figure 1h; Figure S10, Supporting Information) and corresponding UV–vis spectroscopic data (Figure S11, Supporting Information). Calculations determined the electrostatic adsorption efficiency to be 70% (Figure S12, Supporting Information). Importantly, the biorobot exhibited excellent stability, with minimal changes in DLS and polymer dispersion index (PDI) over 10 days in PBS and PBS with 1% FBS (Figure 1i; Figure S13, Supporting Information).

Significantly, the proliferation and viability of bacteria were not affected by Cu_2_O nanoparticles (Figure 1j; Figure S14, Supporting Information). Moreover, the prepared Cu_2_O@*E*_G_ was relatively stable and did not undergo obvious oxidative discoloration (Figure S15, Supporting Information). Cu_2_O nanoparticles incorporation endowed the biorobot with peroxidase (POD)‐like and glutathione peroxidase (GPx)‐like nanozyme activities, enabling both ^•^OH generation and glutathione (GSH) depletion. Subsequently, the intrinsic multienzyme activities of Cu_2_O@*E*_G_ were investigated. POD‐like activity was evaluated using 3,3′,5,5′‐tetramethylbenzidine (TMB) colorimetric assay (Figure 1n). In the presence of H_2_O_2_ and Cu_2_O nanoparticles, the reaction produced a strong blue color and a significant increase in absorbance at 655 nm, indicating robust ^•^OH generation (Figure 1k). Notably, this activity remained unaffected after nanoparticle loading on the bacterial surface. Moreover, higher temperatures and the acidic conditions of the tumor microenvironment (pH = 6.5) enhanced catalytic performance (Figure 1l). To investigate GPx‐mimicking activity, the ability of Cu_2_O@*E*_G_ to deplete intracellular GSH was assessed using the GSH indicator 5,5′‐dithiobis‐(2‐nitrobenzoic acid) (DTNB), which reacts with GSH to produce a yellow compound detectable at 412 nm. Cu_2_O and Cu_2_O@*E*_G_ both significantly reduced DTNB absorbance, indicating effective GSH consumption (Figure 1m,n).

Collectively, these findings demonstrate that Cu_2_O@*E*_G_ exhibits significant POD‐ and GPx‐like catalytic activities, facilitating ^•^OH generation and GSH depletion to create a favorable microenvironment for tumor treatment. These results highlight the potential of our engineered bacterial biorobot as an effective platform for cancer therapy.

### 2.2 In Vitro Evaluation of the Antitumor Activity and GOx Function of Cu_2_O@E_G_

**Figure 2:**
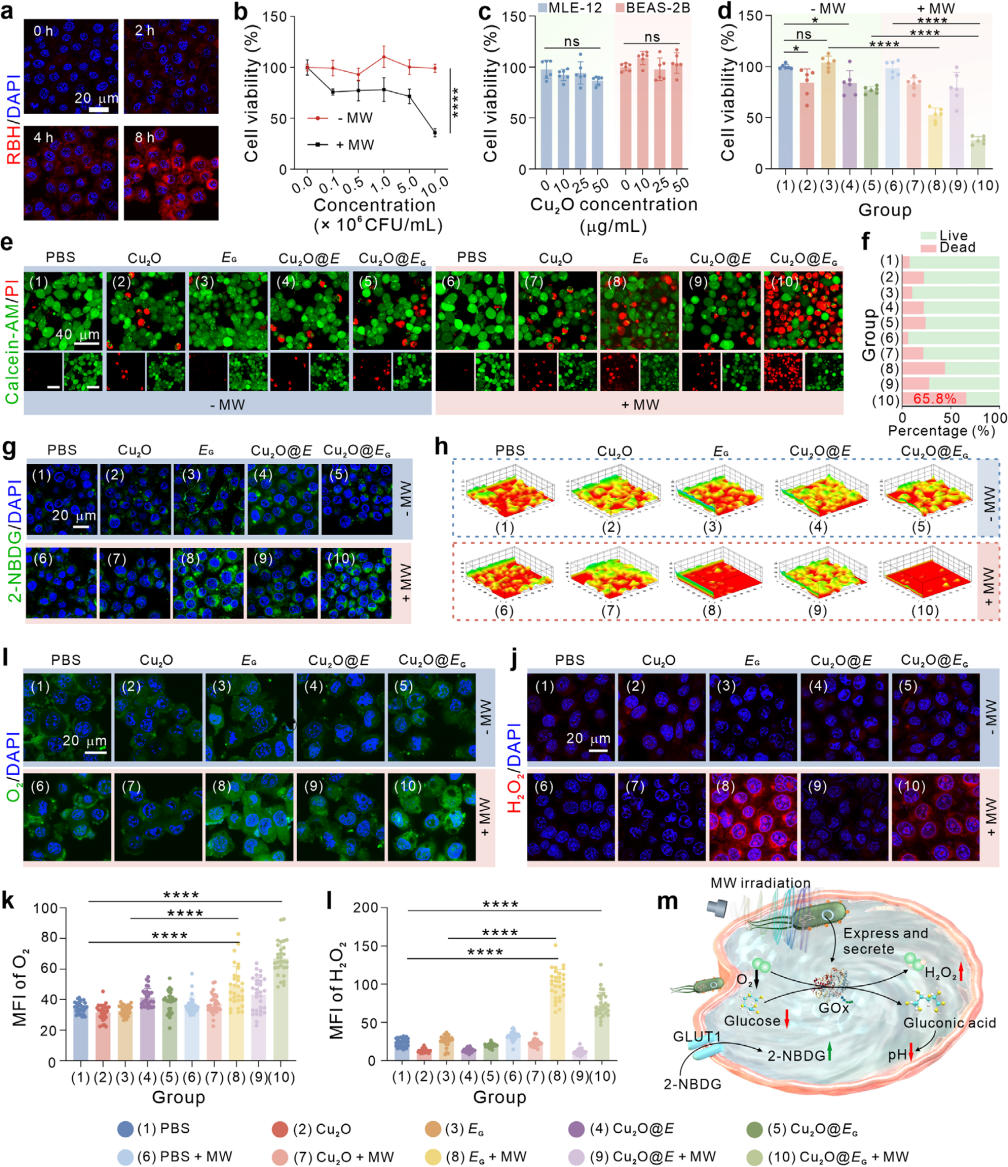
The cancer cell destruction ability and GOx activity of Cu_2_O@*E*_G_ in vitro. a) Endocytosis ability of Cu_2_O@*E*_G_ by 4T1 cells determined by intracellular Cu ions staining. b) Viability assay of 4T1 cells treated with different concentrations of *E*_G_ culture medium under 37 °C and 45 °C conditions (*n* = 6). c) Cytotoxicity of MLE‐12 cells and BEAS‐2B cells exposed to various concentrations of Cu_2_O@*E*_G_ (*n* = 6). d) Cytotoxicity of 4T1 cells exposed to various therapeutic formulations (*n* = 6). Representative CLSM images e) and semi‐quantitative analysis f) of live/dead staining of 4T1 cells treated by various formulations. g) Intracellular 2‐NBDG contents of 4T1 cells in response to various treatments detected by CLSM. h) 3D‐surface plot of intracellular pH CLSM images following treatment with different therapeutic formulations. Intracellular CLSM images and fluorescence intensity quantification of 4T1 cells stained by O_2_ i,k) and H_2_O_2_ j,l), respectively (*n* = 30). m) Schematic illustration of glucose deprivation strategy by Cu_2_O@*E*_G_ under MW irradiation. Data were presented as mean ± S.D. Statistical analysis was calculated by using one‐way analysis of variance with a Tukey's test (****p < 0.0001, *p < 0.1, and ns > 0.05).

To assess the antitumor efficacy of the Cu_2_O@*E*_G_ biorobots, their in vitro performance was systematically evaluated. First, we employed rhodamine B hydrazide (RBH) Cu^2+^ probe staining to monitor intracellular copper ion fluctuations (Figure S16, Supporting Information). As shown in **Figures** **2**a and S17 (Supporting Information), cellular copper ion levels increased in a time‐dependent manner during co‐incubation with Cu_2_O@*E*_G_, confirming progressive uptake. Atomic absorption spectrometer (AAS) results also demonstrated a significant increase in copper ion content in cells treated for 6 h (Figure S18, Supporting Information). Subsequently, we systematically deciphered the cellular uptake mechanism of Cu_2_O@*E*_G_ nanoparticles using a combination inhibitor strategy (Figure S19, Supporting Information). Flow cytometry (FCM) quantification revealed that treatment groups with chlorpromazine (clathrin‐dependent endocytosis inhibitor), methyl‐β‐cyclodextrin (caveolin‐dependent endocytosis inhibitor), and filipin (cholesterol inhibitor) significantly inhibited internalization efficiency, while, in contrast, cytochalasin D (macropinocytosis inhibitor) and wortmannin (phagocytosis and macropinocytosis inhibitor) treatment groups showed no significant effects. These results definitively confirmed that the nanoparticles primarily undergo cellular transport through both the clathrin‐mediated endocytic pathway and caveolin‐dependent lipid raft‐mediated pathway. Confocal microscopy analysis with dual‐color fluorescent labeling (Cy5‐Cu_2_O/FITC‐*E*_G_) demonstrated significant subcellular co‐localization (Pearson's coefficient r = 0.76 ± 0.04) of Cu_2_O@*E*_G_ biohybrids during intracellular trafficking, where their spatial coupling characteristics validated the structural integrity of the composite system throughout cellular transport processes (Figure S20, Supporting Information). In addition, we employed multiplex fluorescence colocalization analysis to delineate the spatiotemporal localization dynamics of Cu_2_O@*E*_G_. The quantitative profiling revealed predominant lysosomal targeting at 2 h, with subsequent time‐dependent lysosomal escape and cytoplasmic redistribution observed at 4 h (Figures S21 and S22, Supporting Information). Collectively, these findings demonstrate that Cu_2_O@*E*_G_ nanoparticles achieve efficient intracellular delivery through lysosomal escape mechanisms, effectively bypassing endolysosomal sequestration. Then, the cytotoxicity of Cu_2_O@*E*_G_ toward tumor cells was assessed using cell counting kit‐8 (CCK‐8) assay. As expected, *E*_G_ alone exhibited excellent biocompatibility with mouse breast cancer 4T1 cells. However, under MW stimulation, *E*_G_ displayed significant cytotoxicity, with a concentration‐dependent effect (Figure 2b). Notably, *E*_G_ at a concentration of 1 × 10^7^ CFU mL^−1^ showed negligible dark toxicity toward normal cells, demonstrating excellent biosafety within the experimental concentration range of Cu_2_O nanoparticles (Figure 2c).

To further investigate, a combination of 1 × 10^7^ CFU mL^−1^ *E*_G_ and 50 µg mL^−1^ Cu_2_O was applied to evaluate the antitumor effects of Cu_2_O@*E*_G_. Upon MW irradiation, the viability of tumor cells treated with Cu_2_O@*E*_G_ decreased markedly to 27.9%, significantly outperforming groups treated with Cu_2_O + MW (82.6%) or *E*_G_ + MW (52.8%) alone (Figure 2d). Live/dead cell staining (Figure 2e,f) further confirmed that Cu_2_O@*E*_G_ + MW induced the highest cell mortality among all treatment groups. These results highlight the synergistic therapeutic efficacy of Cu_2_O@*E*_G_ under MW stimulation, surpassing the effects of the individual components. This novel approach demonstrates promising biocompatibility and substantial antitumor efficacy, offering valuable insights for future research.

Next, the underlying mechanism of tumor cell death induced by Cu_2_O@*E*_G_ was investigated. The enzymatic activity of GOx depletes glucose, compelling tumor cells to increase extracellular glucose uptake to meet metabolic demands. To visualize this phenomenon, fluorescent d‐glucose analogues were used to monitor glucose uptake in different treatment groups. As shown in Figure 2g, the *E*_G_ + MW and Cu_2_O@*E*_G_ + MW groups exhibited significantly elevated fluorescence intensities, indicating nutrient‐deprivation stress with upregulated glucose uptake by tumor cells.

The introduction of GOx results in the rapid oxidation of glucose to gluconic acid, resulting in localized acidification and substantial H_2_O_2_ production. Fluorescence intensity measurements using a pH‐sensitive probe (BCECF) revealed marked acidification in the *E*_G_ + MW and Cu_2_O@*E*_G_ + MW treatment groups due to gluconic acid accumulation (Figure 2h). Simultaneously, intracellular oxygen (O_2_) levels were evaluated using the [Ru(dpp)_3_]Cl_2_ dye, which fluoresces upon O_2_ interaction (Figure 2i,k). Enhanced green fluorescence in the *E*_G_ + MW and Cu_2_O@*E*_G_ + MW groups indicated a notable reduction in intracellular O_2_ levels, suggesting partial reoxygenation of the tumor microenvironment and evidence of tumor metabolic reprogramming. In parallel, intracellular H_2_O_2_ levels were significantly elevated in response to MW stimulation in both *E*_G_ and Cu_2_O@*E*_G_ treatment groups (Figure 2j,l). However, the Cu_2_O@*E*_G_ + MW group displayed slightly lower H_2_O_2_ fluorescence compared to the *E*_G_ + MW group, likely due to the peroxidase‐like activity of Cu_2_O, which consumes H_2_O_2_.

In conclusion, these findings demonstrate that under MW stimulation, Cu_2_O@*E*_G_ triggers a cascade of reactions involving glucose metabolism, acidification, and H_2_O_2_ production via Gox (Figure 2m). This mechanistic insight underscores the therapeutic potential of Cu_2_O@*E*_G_ for cancer treatment and provides a foundation for further investigations.

### 2.3 Synthetic Therapeutic Mechanism of Cu_2_O@*E*_G_ In Vitro

**Figure 3:**
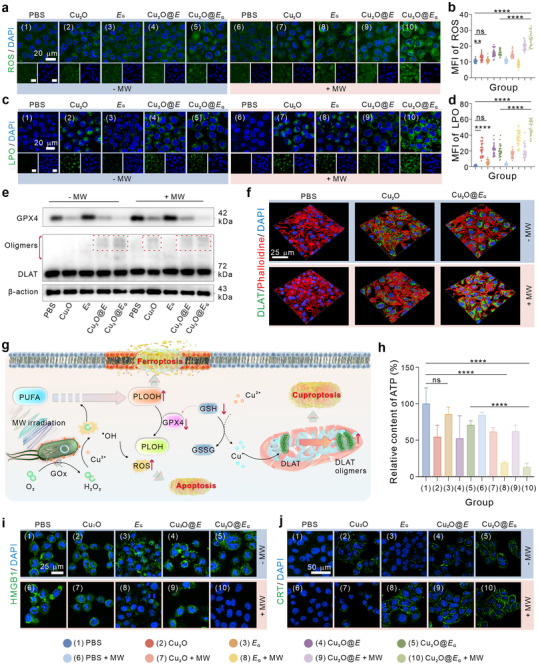
The synthetic therapeutic mechanism of Cu_2_O@*E*_G_ in vitro. Visualization and quantitative analysis of cellular ROS a,b) and LPO c,d) changes induced by the various treatments under confocal microscopy (*n* = 30). f) Visualization of cellular DLAT oligomerisation in 4T1 cells after different treatments. g) Schematic representation of ferroptosis and cuproptosis pathways activated in cells by Cu_2_O@*E*_G_ combined with MW treatment. h) Intracellular ATP content across different treatment groups (*n* = 3). Visualization of cellular HMGB1 i) and CRT j) in 4T1 cells after different treatments. Data were presented as mean ± S.D. Statistical analysis was calculated by using one‐way analysis of variance with a Tukey's test (****p < 0.0001, **p < 0.01, and ns > 0.05).

The prepared biorobot, Cu_2_O@*E*_G_, was designed to induce and enhance tumor cell ferroptosis and curproptosis through the introduction of Cu_2_O nanoparticles. First, the GOx‐mediated catalytic reaction elevated intracellular H_2_O_2_ levels, addressing the substrate limitation for generating ^•^OH via the Cu_2_O‐mediated Fenton‐like reaction (Figure S23, Supporting Information). Analysis of **Figure** **3**a,b revealed a significant increase in intracellular ROS levels in the *E*_G_ + MW group compared to the blank and *E*_G_ groups, primarily due to H_2_O_2_ production. Notably, ROS levels were 1.92‐ and 2.83‐fold higher in the Cu_2_O@*E*_G_ + MW‐treated group than in the Cu_2_O or *E*_G_ + MW‐treated groups, respectively. This excessive ROS accumulation established a conducive environment for apoptosis and ferroptosis. However, elevated endogenous GSH can deplete ROS, mitigating oxidative stress and limiting the efficacy of ROS‐mediated tumor therapies.^[^ ^21^ ^]^ Thus, we investigated GSH levels under Cu_2_O@*E*_G_ treatment. As shown in Figure S24 (Supporting Information) Cu_2_O@*E*_G_ effectively depleted GSH due to the incorporation of Cu_2_O nanoparticles, disrupting intracellular redox homeostasis and enhancing the therapeutic potential of ROS‐related mechanisms.

Next, apoptosis levels were assessed under various treatments. Flow cytometry analysis demonstrated significantly higher apoptosis rates in the Cu_2_O@*E*_G_ + MW group (Figure S25, Supporting Information). In a typical ferroptosis scenario, cellular lipid peroxidation (LPO) increases due to ROS.^[^ ^22^ ^]^ Using Liperfluo fluorescence probes, we observed substantial LPO accumulation in the Cu_2_O@*E*_G_ + MW‐treated group, highlighting pronounced ferroptosis induced by the cascade reaction (Figure 3c,d). However, the glutathione peroxidase 4 (GPX4)/GSH system, a critical antioxidant defense, converts phospholipid hydroperoxides (PLOOH) into nontoxic phospholipid alcohols (PLOH), thus mitigating LPO and ferroptosis.^[^ ^22^, ^23^ ^]^ Western blot analysis revealed significant downregulation of GPX4 expression in cells treated with Cu_2_O and Cu_2_O@*E*_G_, indicating GSH depletion caused by the catalytic activity of Cu_2_O (Figure 3a).

Cuproptosis, a recently characterized form of regulated cell death, involves copper‐ion overload and the aggregation of lipoylated dihydrolipoamide S‐acetyltransferase (DLAT).^[^ ^24^ ^]^ Typically, Cu^+^ ions can directly bind to terminal cysteine residues of lipoylated DLAT, inducing its aggregation.^[^ ^25^ ^]^ Meanwhile, intracellular Cu^2+^ ions can be reduced cytotoxic Cu^+^ state via ferredoxin 1 (FDX1).^[^ ^26^ ^]^ Western blotting and immunofluorescence staining revealed DLAT oligomerization in Cu_2_O and Cu_2_O@*E*_G_‐treated groups, attributable to copper‐ion activity (Figure 3e,f). Treatment with the copper‐chelating agent (tetrathiomolybdate, TM) reduced DLAT aggregation, as shown in Figure S26 (Supporting Information). Collectively, these results illustrate the multifaceted cell demise mechanisms induced by Cu_2_O@*E*_G_ (Figure 3g).

Given the demonstrated efficacy of Cu_2_O@*E*_G_ in inducing apoptosis, ferroptosis, and cuproptosis, we hypothesized that it might trigger immunogenic cell death (ICD) in tumor cells and activate antitumor immunity. During ICD, cells release damage‐associated molecular patterns (DAMPs, such as calreticulin (CRT), high mobility group box 1 (HMGB1), and adenosine triphosphate (ATP).^[^ ^27^ ^]^ These signals promote dendritic cell (DC) maturation and antigen presentation, essential for antitumor immune responses.^[^ ^28^ ^]^ We evaluated ICD hallmarks in 4T1 cells after various treatments. Notably, intracellular ATP levels were significantly reduced in the Cu_2_O@*E*_G_ + MW group, likely due to the combined effects of GOx and ICD (Figure 3h). Similarly, intracellular HMGB1 levels decreased substantially following Cu_2_O@*E*_G_ + MW treatment, a hallmark of ICD progression (Figure 3i). Furthermore, immunofluorescence staining showed intense CRT fluorescence on the cell membrane in the Cu_2_O@*E*_G_ + MW group, indicating robust CRT exposure post‐treatment (Figure 3j).

### 2.4 Transcriptomic Analysis

**Figure 4:**
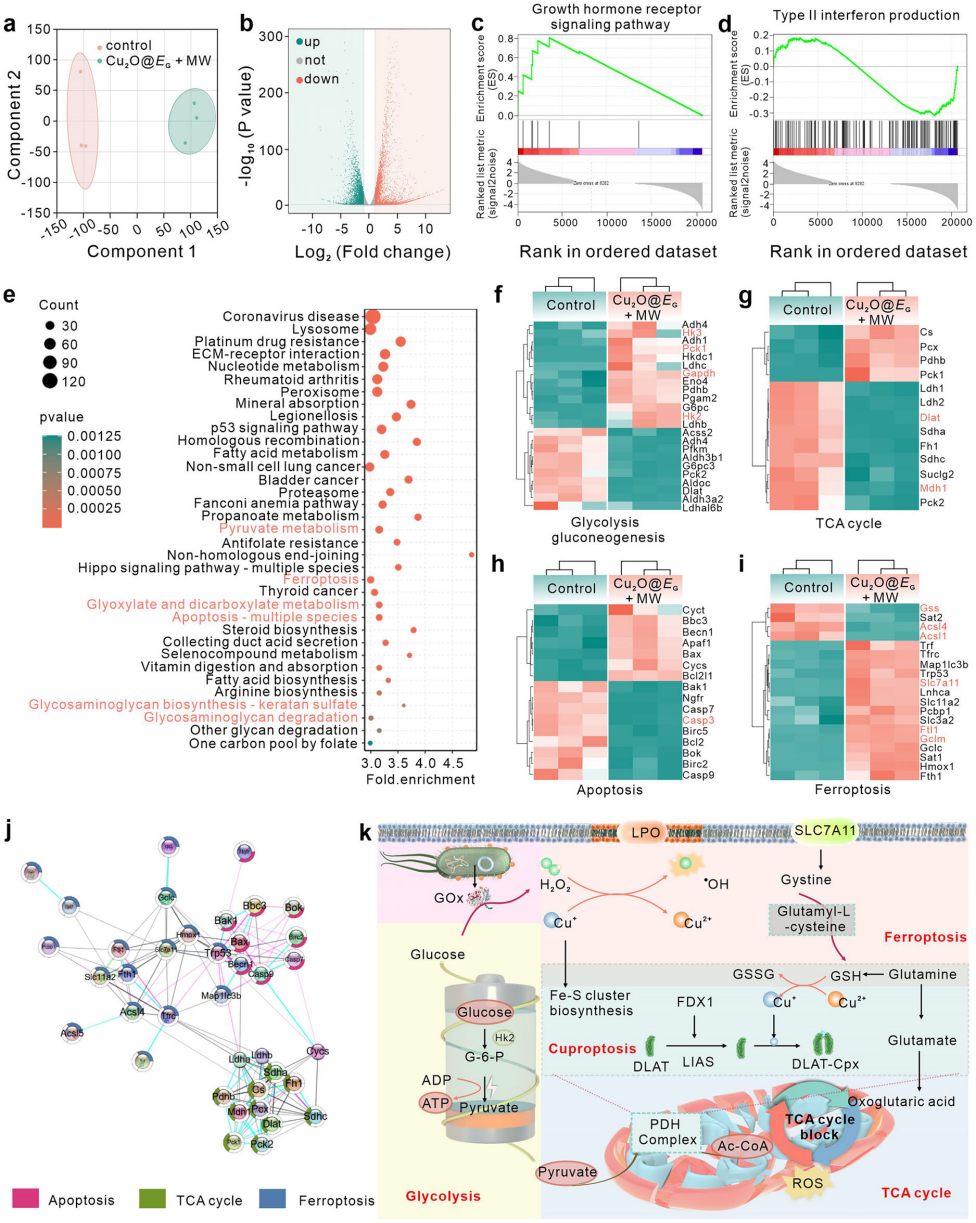
Transcriptomic analysis. a) PCA of the total proteomes from 4T1 cells treated with the different formations. Colors and shapes denote distinct groups, while ellipses highlight clusters of sample types. b) Volcano plot illustrates differentially expressed metabolites relative to the control group. GSEA analysis showed that gene sets of the growth hormone receptor signalling pathway c) and type II interferon production pathway d) were enriched in cells. e) Identification of the top 30 pathways that were significantly upregulated or downregulated following various treatments compared to untreated control cells, based on functional enrichment analysis. Clustering heatmap of differentially expressed genes from glycolysis gluconeogenesis f), TCA cycle g), ferroptosis h), and apoptosis i) pathway between Cu_2_O@*E*_G_ + MW‐treated group and control group. j) Interaction network mapping illustrates the relationships between the TCA cycle, apoptosis, and ferroptosis. k) Summary of the primary antitumor mechanism of Cu_2_O@*E*_G_. *n* = 3 biologically independent cells per group.

To gain deeper insight into the mechanism of Cu_2_O@*E*_G_ biorobots in tumor treatment, RNA sequencing was conducted to analyze gene expression under different intervention conditions. A correlation heatmap (Figure S27, Supporting Information) between control (untreated) and Cu_2_O@*E*_G_ + MW groups revealed strong intragroup correlations and distinct intergroup differences, underscoring the biorobot treatment as a major contributor to genetic variance. Principal component analysis (PCA)further confirmed significant transcriptomic differences, with samples clustering tightly within groups and clear separation observed between groups, demonstrating the high quality and reliability of the data (**Figure** **4**a).

The overall distribution of differentially expressed genes (DEGs) was visualized using a volcano plot (Figure 4b), identifying 2866 upregulated genes (red) and 3738 downregulated genes (green) compared to the control group. Gene Set Enrichment Analysis (GSEA) of all DEGs, based on the Molecular Signature Database, revealed significant activation of immune‐related pathways, particularly those involving growth hormone signaling, indicative of a robust immune response elicited by Cu_2_O@*E*_G_ biorobots in 4T1 cells (Figure 4c,d). To further elucidate the underlying mechanisms, the Kyoto Encyclopedia of Genes and Genomes (KEGG) pathway enrichment analysis highlighted substantial impacts on metabolic pathways. As displayed in Figure 4e, notable changes were observed in glucose metabolic pathways, such as pyruvate metabolism and glyoxylate and dicarboxylate metabolism, as well as in pathways associated with apoptosis, ferroptosis, and multiple‐species apoptosis pathways. A heatmap of DEGs related to glyoxylate metabolism, the tricarboxylic acid (TCA) cycle, apoptosis, ferroptosis, and cuproptosis provided a visual representation of gene expression patterns linked to these pathways (Figure 4f–i). These findings were supported by protein–protein interaction network diagrams, illustrating the interplay between glucose metabolism‐associated TCA cycle due to and patterns of apoptosis and ferroptosis (Figure 4j). Transcriptome sequencing revealed that the combined treatment with Cu_2_O@*E*_G_ and MW significantly impacted multiple molecular pathways, including genes and signalling networks.

These findings offer valuable insights into the intricate changes in the intricate changes in gene expression and pathway dynamics induced by the treatment, particularly highlighting the disruption of the TCA cycle due to glucose metabolism intervention and its association with cuproptosis and ferroptosis (Figure 4k).

### 2.5 In Vivo Distribution and Behavior of Cu_2_O@*E*_G_ in Mice

**Figure 5:**
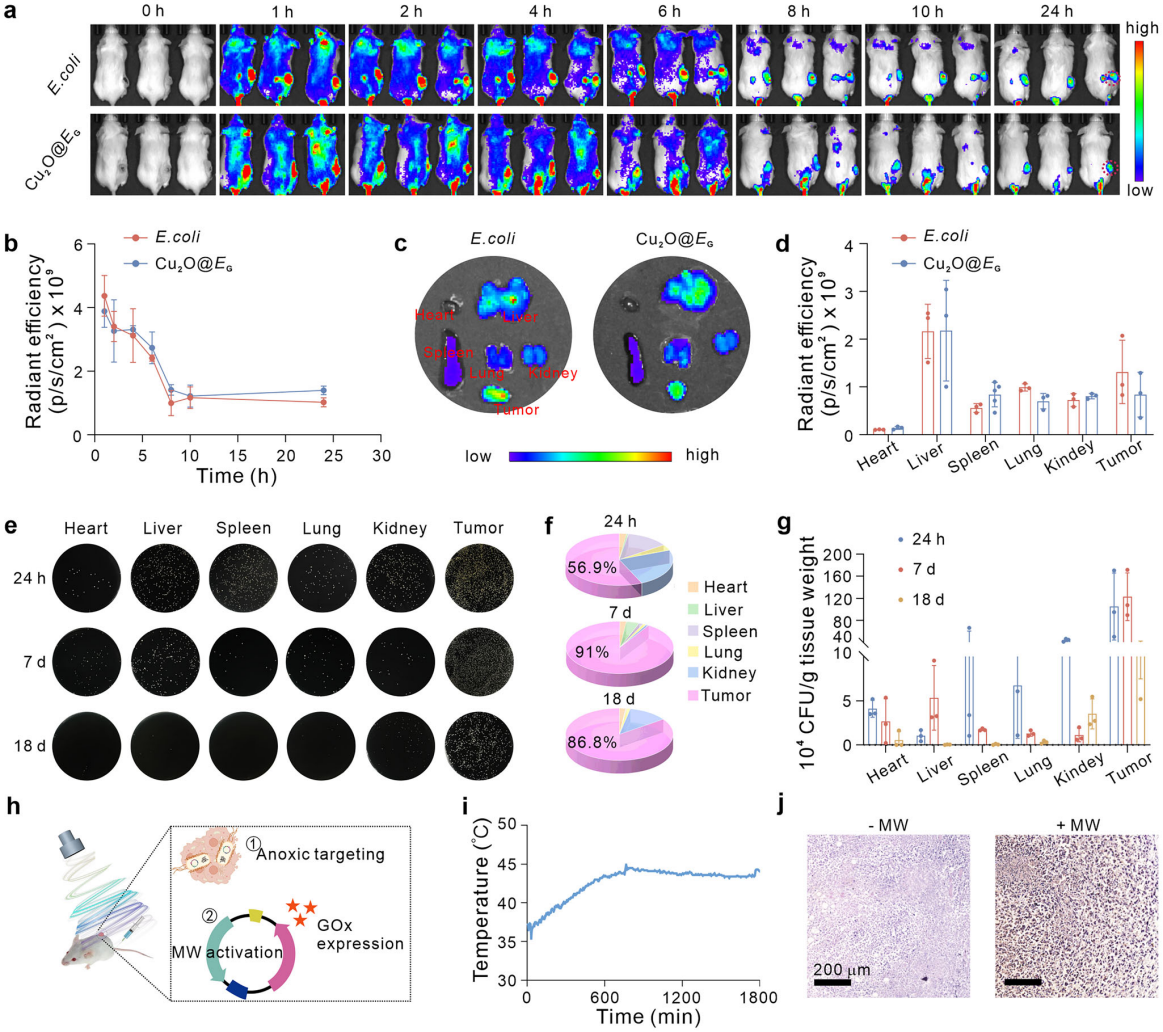
In vivo distribution and behaviours of bacteria in mice. a) In vivo real‐time imaging of tumor‐bearing mice following intravenous injection of different therapies (*n* = 3). b) Mean ICG fluorescence intensities at the tumor site at different time points post‐injection. Ex vivo IVIS images c) and quantitative analysis d) of the major organs (heart, liver, spleen, lung, kidney) and tumor, harvested from the mice after 24 h post‐injection (*n* = 3). Photographs e) and quantitative results f,g) of solid LB agar plates showing bacterial colonization in tumors and major organs collected from tumor‐bearing mice at various time points following intravenous administration of Cu_2_O@*E*_G_ (*n* = 3). h) Schematic representation of the action of the *E*_G_ in tumor‐bearing mice. i) Warming curves of tumor sites in mice under MW irradiation. j) His tag‐stained tumor slices for different treatments. Data were presented as mean ± S.D.

Bacteria are known to preferentially accumulate in tumors due to their affinity for anaerobic environments. To evaluate the tumor‐targeting capabilities of engineered bacteria, indocyanine green (ICG)‐labeled *E. coli* and ICG‐labeled Cu_2_O@*E*_G_ biorobots (Figure S28, Supporting Information). Fourier Transform infrared spectroscopy (FTIR) analysis revealed characteristic absorption peaks of free ICG at 1600 cm^−1^ (aromatic C═C stretching) and 1040 cm^−1^ (symmetric S═O stretching from sulfonate groups) (Figure S29, Supporting Information). Upon formation of the *E. col*i@ICG hybrids, the intensity of the aromatic ring vibration peak was significantly reduced or completely disappeared, suggesting that ICG's hydrophobic benzoindole rings insert into a hydrophobic region of the bacterial membrane.^[^ ^29^ ^]^ 1 × 10^7^ CFU bacteria (contains 100 µg ICG) were intravenously injected into 4T1 tumor‐bearing mice and imaged at various time points using an IVIS Spectrum imaging system. As shown in **Figure** **5**a,b, the *E. coli* demonstrated efficient tumor‐homing ability, with strong fluorescence observed in the 4T1 tumor at 6 h post‐injection. In the results, the fluorescence signal at the tumor site exhibited a significant attenuation after 6 h. This phenomenon is likely attributable to the detachment of ICG from the *E. coli* surface within the mildly acidic tumor microenvironment, a mechanism further corroborated by our in vitro ICG release kinetics assay (Figure S30, Supporting Information). Notably, the surface loading of Cu_2_O nanoparticles did not affect this targeting ability. At 24 h post‐injection, fluorescence imaging of harvested tumors and major organs (heart, liver, spleen, lung, and kidney) revealed significant fluorescence in the liver, a major metabolic organ, while other organs displayed no notable enrichment. Both the *E. coli*‐treated and Cu_2_O@*E*_G_‐treated groups exhibited strong fluorescence signals in tumors (Figure 5c,d).

To further examine the biodistribution and colonization of the Cu_2_O@*E*_G_ biorobot, tumors and major organs were collected at 24 h, 7 days, and 18 days post‐injection. Organs were homogenized, serially diluted with PBS, and plated on solid Luria‐Bertani (LB) agar. Consistent with ex vivo imaging, the biorobot predominantly localized to tumor sites at 24 h, with high levels of colonization persisting at 7 and 14 days, supporting sustained therapeutic effects (Figure 5e–g). By 18 days, the biorobot was cleared from all organs. Pharmacokinetic analysis of Cu_2_O@*E*_G_ biorobot based on blood copper ion levels revealed a rapid absorption profile, with peak plasma concentration (C_max_) achieved at 5 min post‐administration and a terminal half‐life (t_1/2_) calculated to be 118 min (Figure S31, Supporting Information). These findings suggest that biorobot exhibits rapid systemic absorption into the bloodstream, enabling tumor‐specific accumulation, while maintaining efficient systemic clearance via hepatic and renal pathways. Furthermore, we analyzed copper ion distribution in mice. The results demonstrated that mice treated with biorobot for 24 h exhibited a 2.7‐fold increase in copper ion content within tumor tissues compared to the untreated group. Notably, elevated copper levels persisted in tumor tissues even 18 days post‐administration, which may enhance sustained therapeutic efficacy (Figure S32, Supporting Information). Additionally, hemolysis experiments showed that *E*_G_ (1 × 10^7^ CFU mL^−1^) with various concentration of Cu_2_O nanoparticles did not cause hemolysis of red blood cells (Figure S33, Supporting Information). To further evaluate the biosafety of Cu_2_O@*E*_G_, healthy Balb/c mice were intravenously administered 1 × 10^7^ CFU mL^−1^ Cu_2_O@*E*_G_ (Figure S34a, Supporting Information). Over a 60‐day evaluation period, no significant changes in body weight, liver, or kidney function were observed, and histological examination revealed no pathological damage in tissue sections (Figure S34b–n, Supporting Information). These findings underscore the excellent biocompatibility of Cu_2_O@*E*_G_ and mitigate concerns about the safety of bacterial therapies.

These results demonstrate that the Cu_2_O@*E*_G_ biorobot exhibits precise tumor targeting in the hypoxic tumor microenvironment and is effectively cleared from other organs. Finally, we investigated the behavior of biorobot expression under MW stimulation (Figure 5h). Figure 5i shows that MW irradiation increased tumor temperature to 45 °C within 10 min, maintaining it between 42 and 45 °C with intermittent exposure. This indicates that MW irradiation can be used as a remote tool to regulate therapeutic expression by engineered bacteria. Then, we verified the expression of GOx in tumor‐bearing mice, and as shown, GOx was only expressed in response to MW stimulation (Figure 5j).

### 2.6 **In** Vivo Antitumor Activities **of Cu_2_O@*E*_G_**

**Figure 6:**
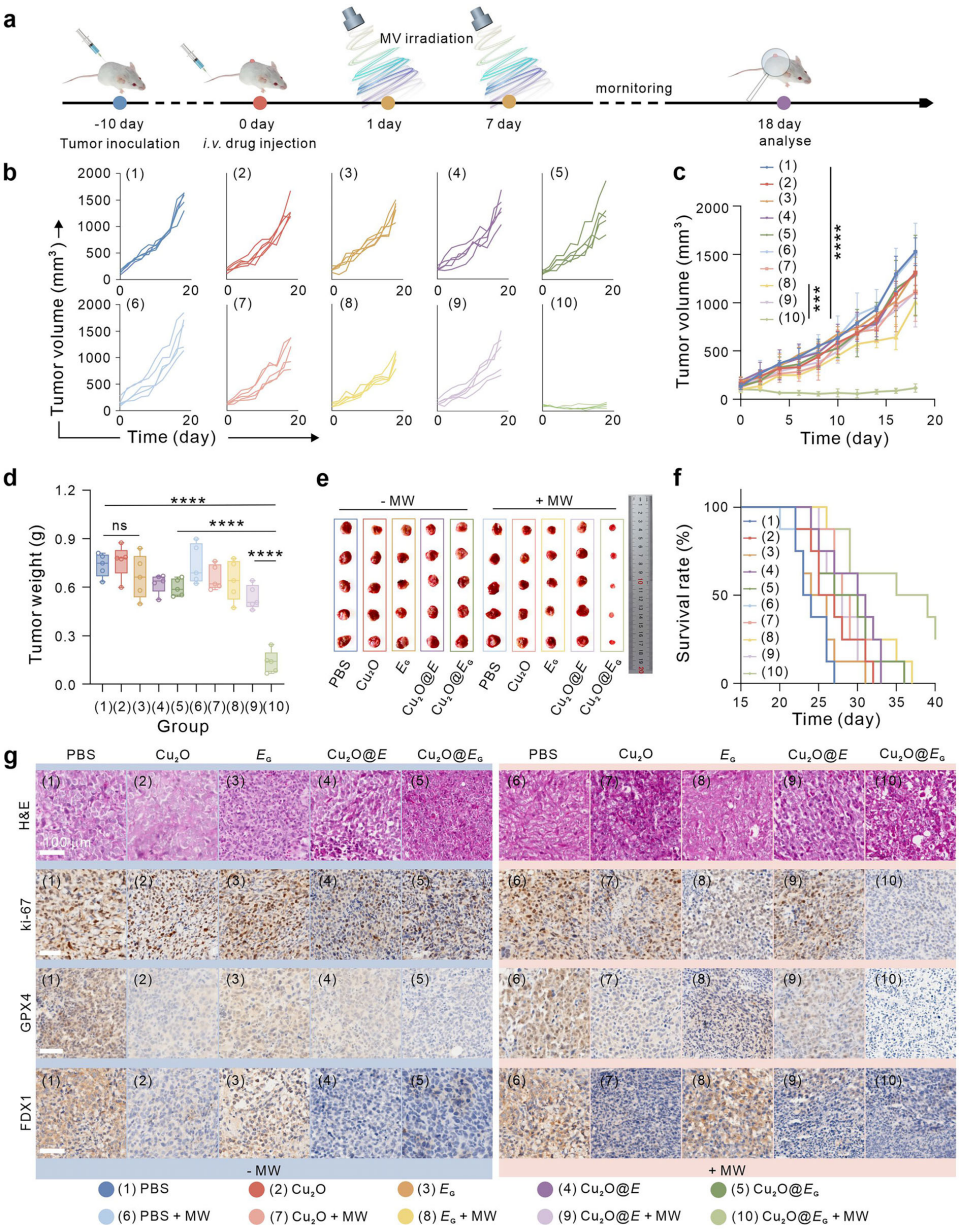
The antitumor activities of biorobot in vivo. a) Schematic illustration of the treatment design. b) Tumor growth curve of tumor‐bearing mice treated with different treatments. c) Tumor growth curve of tumor‐bearing mice treated with different treatments. d) Weights of the tumors extracted from mice in varied groups at the end of therapy. e) Photograph of excised 4T1 tumors after various treatments. f) Survival curves for different treatment groups. g) H&E‐stained, ki‐67‐stained, GPX4‐stained and FDX1‐stained tumor slices for each group. Data were presented as mean ± S.D with *n* = 5 biologically independent animals per group. Statistical analysis was calculated by using one‐way analysis (b) or two‐way (c) analysis of variance with a Tukey's test (****p < 0.0001, ***p < 0.001, and ns > 0.05).

Building on the promising in vitro antitumor performance, the therapeutic efficacy of Cu_2_O@*E*_G_ in vivo was evaluated a 4T1 tumor‐bearing mouse model, following the treatment regimen outlined in **Figure** **6**a. Mice were treated with PBS, Cu_2_O, *E*_G_, Cu_2_O@*E* or Cu_2_O@*E*_G_, with MW irradiation (triangle wave, 15 W, 30 min) applied at 24 h and 7 days post‐injection to induce GOx biosynthesis. Tumor volume and body weights were monitored every 2 days for 18 days (Figure 6b,c; Figure S35, Supporting Information).

Throughout the treatment, no significant body weight fluctuations were observed in any group, indicating minimal adverse effects. Tumors in the PBS and MW‐only groups grew consistently, whereas the Cu_2_O@*E*_G_ + MW group demonstrated the most effective tumor inhibition, achieving an 81.5% suppression rate (Figure S36, Supporting Information). Tumor weights further validated these results (Figure 6d,e), and survival rates in the Cu_2_O@*E*_G_ + MW group significantly increased (Figure 6f). Similar results were observed in the highly invasive B16‐F10 tumor model, further confirming the antitumor efficacy (Figure S37, Supporting Information).

Hematoxylin and Eosin (H&E) histological analyses revealed severe tumor damage in the Cu_2_O@*E*_G_ + MW group (Figure 6g), underscoring its potent therapeutic effects. Importantly, no pathological abnormalities were detected in major organs across all treatment groups (Figure S38, Supporting Information), indicating low systemic toxicity. Immunohistochemistry analysis showed significant downregulation GPX4 expression in the Cu_2_O@*E*_G_ + MW group after 18 days, weakening the tumor's antioxidant system and promoting ferroptosis (Figure 6g). Concurrently, the expression of the cuproptosis‐associated marker FDX1 was markedly suppressed, with minimal FDX1‐positive signals observed, highlighting the synergistic activation of ferroptosis and cuproptosis (Figure 6g).

### 2.7 Cu_2_O@*E*_G_ Augmented Antitumor Immune Responses

**Figure 7:**
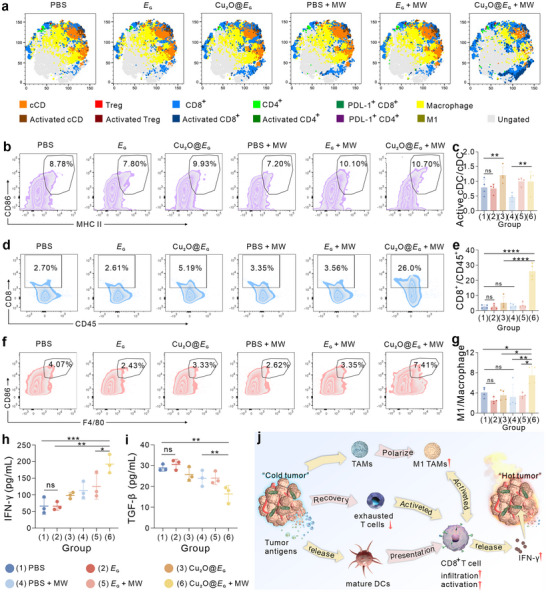
Biorobot augmented antitumor immune response. a) t‐SNE plot of the major cell populations identified. Representative flow cytometric plots b) and statistical analysis c) of mature DCs upon the indicated treatments by flow cytometry. Representative flow cytometric plots d) and statistical analysis e) of CD8^+^ T cells upon the indicated treatments. Representative flow cytometric plots f) and statistical analysis g) of M1‐like macrophages upon different treatments. *n* = 4 biologically independent animals per group. Cytokine IFN‐γ h) and TGF‐β i) secretion upon different treatments. (*n* = 3). j) Schematic representation of Cu_2_O@*E*_G_ mediated immunotherapy. Data are presented as mean ± standard error of the mean with *n* = 4 biologically independent animals per group. Statistical analysis was calculated by using one‐way analysis of variance with a Tukey's test (****p < 0.0001, ***p < 0.001, **p < 0.01, *p < 0.1, and ns > 0.05).

Encouraged by the promising antitumor efficacy of biorobot observed both in vitro and in vivo, we further assessed its therapeutic potential in 4T1 tumor‐bearing mice. Tumors were harvested following various treatments to evaluate the infiltration of antitumor immune cells using flow cytometry (**Figure** **7**a; Figure S39, Supporting Information). Dendritics cells (DCs), essential antigen‐presenting cells that bridge innate and adaptive immunity,^[^ ^10d^ ^]^ were analyzed for maturation (CD80^+^CD86^+^). The Cu_2_O@*E*_G_ + MW group exhibited a significant increase in mature DCs, from 8.78% in the PBS group to 10.7% (Figure 7b,c), likely due to enhanced tumor antigen release resulting from multimodal tumor cell death.

Next, we investigated the infiltration and activation of CD8^+^ T cells, which directly mediate tumor cell killing.^[^ ^30^ ^]^ The Cu_2_O@*E*_G_ + MW treatment markedly increased CD8^+^ T cell infiltration, raising the percentage from 2.7% in the PBS group to 26.0%, significantly surpassing the 5.19% observed in the Cu_2_O@*E*_G_ groups (Figure 7d,e; Figure S40, Supporting Information). Notably, this treatment induced the most robust immune response, as evidenced by increased CD8^+^ T cell activation and a reduced proportion of PD‐1^+^CD8^+^ T (an exhaustion marker) (Figure S41, Supporting Information).

Flow cytometry analysis of tumor‐associated macrophages (TAMs) further revealed a substantial increase in M1 macrophages (CD86^+^F4/80^+^) in the Cu_2_O@*E*_G_ + MW compared to PBS, Cu_2_O@*E*_G_ alone, and MW alone. This finding indicates effective TAM polarization from the immunosuppressive M2 phenotype to the pro‐inflammatory M1 phenotype (Figure 7f,g). Serum cytokine levels, measured by ELISA, provided additional evidence of immune activation. Interferon‐γ (IFN‐γ), a key cytokine produced by CD8^+^ cytotoxic T lymphocytes to suppress tumor initiation, growth, and metastasis, was significantly elevated in the Cu_2_O@*E*_G_ + MW group (Figure 7h). Conversely, transforming growth factor beta (TGF‐β) levels were markedly reduced, reflecting inhibition of tumor‐promoting pathways (Figure 7i).

Collectively, these results demonstrate that the Cu_2_O@*E*_G_ biorobot enhances tumor immunotherapy under MW stimulation. This antitumor strategy effectively induces apoptosis, ferroptosis, and cuproptosis, thereby promoting the in situ presentation of tumor‐associated antigens (TAAs) and stimulating robust antitumor immune responses. The released TAAs drive DC maturation, initiating adaptive immunity via antigen‐presenting cell‐mediated pathways and counteracting the tumor immunosuppressive microenvironment. Enhanced DC maturation further facilitates T cell proliferation, significantly increasing tumor‐infiltrating CD8^+^ T cells and transforming “cold” tumors into more immunologically active “hot” tumors (Figure 7j).

## 3 Conclusion

By the 1870s, the presence of bacteria in tumors was recognized, promoting exploration of their therapeutic potential in cancer treatment.^[^ ^31^ ^]^ Bacterial therapy offers unique advantages, including specific immune recognition and tumor cell elimination through anaerobic localization, thereby bypassing immune surveillance. Advances in synthetic biology have enabled the engineering of bacteria to release therapeutic agents under precise conditions, enhancing both efficacy and control. Additionally, this approach synergizes with other therapies, amplifying targeted effects while minimizing immune evasion, ultimately improving therapeutic precision.

In this study, we developed a biorobot for multimodal tumor treatment. This system integrates MW‐activated mechanism to control the expression of GOx in response to localized stimuli. This precise regulation addresses key limitations of traditional cancer therapies, particularly regarding the specificity and safety of therapeutic gene expression at tumor sites. Upon activation, GOx catalyzes a reaction that depletes glucose and generates H_2_O_2_, initiating starvation therapy and enhancing ROS production. This effect is further amplified by Cu_2_O nanoparticles, which elevate intracellular copper ion levels and induce cuproptosis. The combination of these cytotoxic pathways maximizes therapeutic efficacy within a single platform. As demonstrated in Figure S34 (Supporting Information) the biorobot achieved an 81% tumor inhibition rate in 4T1 tumor‐bearing mice, underscoring its potent antitumor effects. The induced cell death released substantial tumor antigens, which elicited a robust immune response characterized by significant CD8^+^ T cell activation and an increase in M1 macrophages. Importantly, the treatment reduced exhausted T cell populations, effectively transforming immunologically “cold” tumors into active “hot” tumors. This multifaceted approach disrupts cancer cell metabolism while enhancing tumor immunogenicity, providing a potential synergy with existing immunotherapies.

Translating bacterial therapy from preclinical models to clinical application requires addressing several critical factors. These include the precise localization of bacteria, ensuring host biosafety, optimizing drug release timing and dosage, and effective regulation and clearance of the bacterial agents. To address these concerns, we conducted long‐term toxicity studies in mice. No significant abnormalities were observed in routine blood analyses, liver and renal function indices, or histopathological examination of major organs in either the biorobot or control groups. Moreover, no signs of over‐infection, such as sepsis, were detected during the treatment or toxicity assessment phases. The use of noninvasive MW irradiation for tumor site‐specific activation further enhanced spatiotemporal precision compared to constitutive promoters, mitigating risks associated with systemic undifferentiated expression.

In conclusion, we have developed a programmable bioactive therapeutic platform that leverages MW activation to induce multiple cell death pathways and enhance tumor immunogenicity. This innovative biorobot exemplifies the potential of integrating synthetic biology with nanomaterials to create precisely controlled therapeutic solutions. While challenges remain for clinical translation, our findings lay the groundwork for new strategies in cancer treatment, combining engineered bacteria with advanced therapeutic modalities. Continued exploration of such integrated systems promises to address critical challenges in oncology, potentially revolutionizing cancer therapy and improving patient outcomes.

## 4 Experimental Section

### Materials

Copper acetate, polyvinyl pyrrolidone (PVP, MW 29000), ascorbic acid, cetyltrimethyl ammonium bromide (CTAB), NaOH, fluorescein diacetate, and NaHS were bought from Shanghai Aladdin Biochemical Technology Co., Ltd. GSH assay kit and 1% TMB solution were purchased from Solarbio Science & Technology Co., Ltd. Fetal bovine serum (FBS) was got from PAN. Dulbecco's modified Eagle medium (DMEM) and phosphate buffer solution (PBS) were received from Wuhan Servicebio Technology Co., Ltd. CCK8 kit was received from YENSEN Technology Co., Ltd. 4′,6‐diamidino‐2‐phenylindole (DAPI) was purchased from Beyotime. Liperfluo and Annexin V‐FITC/PI apoptosis detection kit were gained from Dojindo Molecular Technologies, Inc. Beta actin (β‐actin), HMGB1, CRT, DLAT, FDX1, GPX4, HRP‐conjugated Affinipure Goat Anti‐Rabbit IgG (H+L), and HRP‐conjugated Affinipure Goat Anti‐Mice IgG antibodies were obtained from Proteintech.

### Characterization

TEM images were measured on Hitachi HT7700 transmission electron microscope. XPS spectra were measured by the Thermo SCIENTIFIC Nexsa a K‐Alpha 1063 instrument (Thermo Fisher Scientific, USA). XRD patterns were acquired by diffractometer (X'Pert PROPANalytical B.V.). DLS were measured on the Malvern Zetasizer Nano ZS (Malvern Instruments, Ltd., Worcestershire, UK). The fluorescent images were conducted by CLSM (FV3000 Olympus, Japan). FCM (FACSymphony A5, BD Bioscience) was used for immune response analysis. UV–vis absorption spectra were taken on an 8000S spectrometer (METASH). The microplate reader (Thermofisher, Multiskan Sky) was used to detect cell viability, GSH, and TMB catalysis.

### Strain and Plasmids

A genetic sequence comprising an N‐terminal OmpA secretion signal sequence, the GOx gene sourced from *Aspergillus niger*, and a His‐tag was integrated into a pBV220 plasmid using EcoRI and BamHI enzyme digestion and ligation reactions, leading to the creation of the GOX‐expressing plasmid (pBV220‐GOx‐His). Subsequently, the resultant recombinant plasmid was introduced into *E. coli* T1 competent cells through a chemical transformation procedure. In parallel, a control plasmid expressing mCherry (pBV220‐mCherry) was engineered to aid in gene expression detection. Both the pBV220‐GOX‐His and pBV220‐mCherry plasmids underwent chemical transformation into the *E. coli* MG1655 strain following the same outlined protocol. The resulting bacterial solution was plated on LB agar plates containing ampicillin and then left to incubate at 37 °C for 16 h. Following this incubation period, the bacteria were isolated and amplified in LB medium at 37 °C with agitation at 220 rpm overnight. Subsequently, the bacteria underwent a 100‐fold dilution in LB medium and were cultured until reaching an optical density at 600 nm within the range of 0.4–0.6. The bacterial culture was then subjected to incubation at 45 °C for designated durations and subsequently cultured at 37 °C for an additional 16 h in preparation for subsequent experimental procedures.

### Microwave Thermal Performance

The temperature changes in 2 mL of PBS were monitored in a small dish (35 mm × 12 mm) during various durations of exposure to different MW treatment modalities (2.45 GHz, Shengpu Medical Equipment Technology Co., Ltd., China), with temperature readings recorded every second using an infrared thermal imaging camera.

### Thermal‐Induced and Microwave‐Induced mCherry and GOx Expression In Vitro

Initially, to assess the potential for thermal‐induced expression in engineered bacteria, MG1655 cells transformed with the pBV220‐mCherry plasmid at either 37 °C or 45 °C for varying time periods were incubated. Subsequently, the fluorescence of mCherry was examined utilizing Confocal Laser Scanning Microscopy (CLSM) and FCM. Furthermore, Western blot analysis was utilized to confirm the expression of GOx in *E*_G_ upon exposure to thermal and microwave irradiation. Briefly, following incubation at 37 °C, 45 °C, or exposure to microwave irradiation (triangular pulse, 15 W, 2.45 GHz) for 30 min, followed by an additional 24‐h cultivation at 37 °C, both bacterial lysates and culture media were gathered to more precisely evaluate the expression levels of GOx.

### Comparison of Bacterial Viability

After 30 min of treatment at 45 °C or MW irradiation (triangular pulse, 15 W, 2.45 GHz), 1 mL of bacterial culture was collected, stained with FDA, and analyzed for fluorescence intensity using CLSM.

### Synthesis of Cu_2_O Nanoparticles

Cu_2_O nanoparticles were synthesized based on a previous study.^[^ ^19^ ^]^ In brief, 80 mg of copper acetate monohydrate (Cu(Ac)_2_ H_2_O) and 1 g of polyvinylpyrrolidene (PVP, MW K30) were completely dissolved in 30 mL of ethylene glycol. After stirring the mixture at 70 °C for 2 h, 10 mL sodium hydroxide (NaOH, 2 m) was added. After stirring for 30 min, 10 mL ascorbic acid (AA, 26.42 mg mL^−1^) was added. Then, the Cu_2_O was obtained by centrifuging after another 30 min reaction at 70 °C.

### The Preparation of Cu_2_O@CTAB

The resultant Cu_2_O NPs were then dispersed in deionized water (1 mg mL^−1^) and subjected to CTAB (1 mg mL^−1^) surface modification by stirring at room temperature for 6 h. The colloidal suspension was centrifuged at 12 000 rpm for 15 min to remove unbound CTAB, followed by three washing cycles with water. Finally, the CTAB‐modified Cu_2_O NPs (Cu_2_O@CTAB) were lyophilized for 48 h and stored in anhydrous conditions.

### The Preparation of Cu_2_O@E_G_

The Cu_2_O@*E*_G_ was synthesized by electrostatic interaction based on positively charged Cu_2_O@CTAB and negatively charged *E. coli*. Detailly, 2 mL of *E. coli* (≈10^8^ CFU mL^−1^) was mixed with 1 mL of Cu_2_O@CTAB (1 mg mL^−1^) in PBS and further gently shaken at 4 °C for 30 min. Finally, the Cu_2_O@*E*_G_ biorobot was obtained after centrifuging (6000 g, 3 min) and washing with PBS.

### Impact of Cu_2_O Nanoparticles on the Viability of *E. coli*

Bacterial viability was evaluated by FDA staining followed by flow cytometric analysis of FITC‐A channel fluorescence intensity after treatment with Cu_2_O NPs at varying concentrations (0, 50, 80, 100 µg mL^−1^) for 4 and 24 h.

### Measurement of ^•^OH Generation

The production of ^•^OH was quantitatively detected by TMB. Typically, 150 µL water solutions of Cu_2_O NPs, *E. coli*, and Cu_2_O@*E*_G_ (at an equivalent concentration of Cu_2_O NPs) were added into the mixture, which contains 50 µL TMB and 5 µL H_2_O_2_ (20 mM), respectively. After 5 min of reaction, the absorption value of the solution at 652 nm was measured by a microplate reader.

### Measurement of GSH Detection

The GSH content was measured by DTNB at the wavelength of 410 nm. The solutions of Cu_2_O NPs, *E. coli*, and Cu_2_O@*E*_G_ (at an equivalent concentration of Cu_2_O NPs) were mixed separately in GSH (400 µg mL^−1^) and were performed at 37 °C for 12 h. After that, DTNB solution (0.5 mm) was added to detect the remaining GSH. Finally, the UV–vis of the abovementioned samples was recorded.

### Cell Culture

4T1 and B16‐10F cells were cultured in DMEM and 1640 containing 10% fetal bovine serum and 1% penicillin–streptomycin and placed in an incubator at 37 °C with 5% CO_2_.

### Cell Uptake

Cellular uptake behaviors of Cu_2_O@*E*_G_ (with the same concentration of Cu_2_O nanoparticles: 50 µg mL^−1^ and *E. coli*: 1 × 10^7^ CFU mL^−1^) were investigated by CLSM. After being seeded in CLSM dishes and cultured overnight, the 4T1 cells were cocultured with Cu_2_O@*E*_G_ for 0, 2, 8, and 12 h. After that, the cells were washed with PBS several times and strained with Cu ion fluorescence probe (Rhodamine B hydrazide, RBH). Then, the cells were washed with PBS several times to remove the unbound fluorescent dye. Finally, fluorescence of Cu ions was detected by CLSM.

### Detection of Intracellular Copper Ion Levels

4T1 cells were treated with Cu_2_O@*E*_G_ for 6 h, after which the cells were collected, and copper ion content was analyzed using AAS.

### Cellular Uptake Mechanisms

Cellular uptake mechanisms of FITC‐labeled Cu_2_O@*E*_G_ were systematically characterized using a combinatorial inhibitor assay. Briefly, 1 × 10^6^ 4T1 cells were seeded in six‐well plates and allowed to adhere overnight. Cells were pretreated for 2 h with specific endocytosis inhibitors at optimized concentrations: cytochalasin D (1 µm), chlorpromazine (10 µg mL^−1^), methyl‐β‐cyclodextrin (10 mM), wortmannin (100 nM), and filipin (2.5 µg mL^−1^). Following inhibitor pretreatment, cells were incubated with FITC‐ Cu2O@EG for 4 h. Finally, uptake efficiency was quantified by FCM.

### Cu_2_O@E_G_ Dual‐Color Fluorescence Colocalization Analysis

First, a total of 1 × 10^9^ EG were resuspended in 10 µL of FITC solution (2 mg mL^−1^) and incubated under gentle stirring at 4 °C for 30 min. After incubation, cells were washed twice with PBS and resuspended in sterile PBS. Cu_2_O@CTAB nanoparticles (1 mg) were surface‐functionalized with APTES for 2 h. The resulting Cu_2_O@CTAB@APTES particles were incubated with Cy5‐NHS ester overnight at 4 °C with stirring. Unreacted Cy5 was removed via centrifugation, and particles were washed three times with PBS. Cy5‐labeled Cu_2_O@*E*_G_ nanoparticles (50 µg) were mixed with FITC‐labeled *E. coli* (1 × 10^8^ CFU mL^−1^) in PBS at room temperature for 1 h to facilitate electrostatic adsorption. The mixture was then centrifuged to remove free bacteria. Cy5‐labeled Cu_2_O@*E*_G_ nanoparticles were mixed with FITC‐labeled *E. coli* in PBS for 1 h to facilitate electrostatic adsorption. The mixture was then centrifuged to remove free bacteria, and the pellet was resuspended in cell culture medium. The dual‐labeled Cu_2_O@*E*_G_ biohybrid was added to the cells and co‐incubated for 4 h. After washing with PBS, cells were imaged using a CLSM.

### Subcellular Localization of Cu_2_O@E_G_

The intracellular trafficking of Cu_2_O@*E*_G_ was analyzed using CLSM with dual‐channel fluorescence imaging. 4T1 cells were co‐incubated with FITC‐labeled Cu_2_O@*E*_G_ for 2 h. Following incubation, cells were stained with LysoTracker Red or MitoTracker Deep Red. Fluorescent images were subsequently captured using CLSM. Pearson's correlation coefficient (r) was calculated using ImageJ to quantify colocalization.

### Time‐Dependent Lysosomal Escape Analysis

To evaluate lysosomal escape dynamics, 4T1 cells were incubated with FITC‐labeled Cu_2_O@*E*_G_ for 2 or 4 h. Then, cells were stained with LysoTracker Red for 30 min. CLSM imaging was performed to capture spatiotemporal redistribution. Fluorescence intensity profiles were analyzed using ImageJ to quantify lysosomal escape efficiency.

### Cell Viability Assay

The cytotoxicity of various formulations was assessed using the cell counting kit‐8 (CCK‐8) assay. Specifically, 4T1 cells were seeded into 96‐well plates at a density of 1 × 10^4^ cells per well. Then, the old medium was replaced with 100 µL fresh medium containing different concentrations of the samples (PBS, Cu_2_O, *E. coli*, Cu_2_O@*E*, Cu_2_O@*E*_G_ with the same concentration of Cu_2_O nanoparticles: 50 µg mL^−1^ and *E. coli*: 1 × 10^7^ CFU mL^−1^) after the cells were completely attached. After 12 h, the cells were either treated with or without irradiation for 30 min and then further cultured for another 12 h. Following an additional 12‐h incubation period, the cells were washed with PBS several times, and the cell viability was determined using the CCK‐8 assay. The percentage of cell viability was calculated by comparing the absorbance to that of untreated cells. The cytotoxicity of various formulations was assessed using the cell counting kit‐8 (CCK‐8) assay. The same method was used for effects on the viability of normal cells. Bease‐2B cells (human normal lung epithelial cells) and MLE‐12 cells (mouse normal lung epithelial cells) were spread evenly in 96‐well plates and treated with different concentrations of Cu_2_O@*E*_G_ for 24 h of co‐incubation after the cells were fully walled, respectively. And cell viability was assayed with the CCK‐8 kit after 24 h.

### Apoptosis Analysis

For apoptosis analysis, 4T1 cells were seeded into six‐well plates with different formulations (containing the same Cu_2_O nanoparticles: 50 µg mL^−1^ and *E. coli*: 1 × 10^7^ CFU mL^−1^). Then the total cells were collected for flow cytometry‐based Annexin V‐FITC/PI assays to determine the percentage of apoptosis.

### Cellular GSH Detection

The cellular GSH content was detected by the GSH assay kit. In brief, 4T1 cells were seeded in a six‐well plate for 24 h at 37 °C. Then, the cells were treated with PBS, Cu_2_O, *E*_G_, Cu_2_O@*E*, Cu_2_O@*E*_G_, respectively, and incubated for another 12 h. After a further 12 h incubation, the DMEM was removed, and cells were washed with PBS three times. Then, the cells were collected and analyzed by GSH content assay kit according to the published method.

### Live/Dead Staining

The live/dead cell staining was performed using Calcein‐AM and propidium iodide (PI) live/dead viability/cytotoxicity assay kit, following a published protocol. Briefly, 4T1 cells were seeded into CLSM dishes and cultured overnight. Then, the cells were treated with different formulations (PBS, Cu_2_O, *E*_G_, Cu_2_O@*E*, Cu_2_O@*E*_G_ with the same concentration of Cu_2_O nanoparticles: 50 µg mL^−1^ and E. coli: 1 × 10^7^ CFU mL^−1^). After 12 h, followed by treating with or without MW irradiation (triangular pulse, 15 W, 2.45 GHz) for 30 min and further cultured for another 12 h. After that, the cells were stained with Calcein‐AM/PI before conducting CLSM.

### Immunofluorescence Assay

4T1 cells were harvested and seeded at a density of 1 × 10^4^ cells per well in small confocal dishes overnight. Afterward, various therapeutic agents were added separately to the cells. After 24 h of treatment, the original DMEM was removed, and the cells were washed several times with PBS. Then, cell samples were immobilized with 4% PFA and permeabilized with 0.3% Triton X‐100. Following this, the cell samples were incubated with PBS supplemented with 1% BSA for 30 min to block nonspecific binding. Next, the samples were separately incubated with anti‐DLAT, anti‐calreticulin, and anti‐HMGB1antibodies overnight, followed by incubation with secondary antibodies of the same origin at room temperature for 1 h. Finally, the cell samples were stained with DAPI (1 mg mL^−1^) and imaged using CLSM.

### Intracellular H_2_O_2_, O_2_, ROS, and LPO Detection

The cellular content of H_2_O_2_, O_2_, ROS, and LPO was detected using CLSM. 4T1 cells were seeded at a density of 1 × 10^4^ cells/well and incubated with 1 mL DMEM containing different therapeutic agents. Following this, the cells were subjected to treatment with or without microwave irradiation (triangular pulse, 15 W, 2.45 GHz) for 30 min after 12 h. After an additional 12 h of culturing, the cells were washed three times with PBS and then stained Amplex Red (H_2_O_2_ fluorescence probe), Ru(dpp)Cl_2_ (O_2_ fluorescence probe), DCFH‐DA (ROS fluorescence probe), and liperfluo (LPO fluorescence probe) according to a published method, respectively. Subsequently, the cells were washed twice with PBS and then analyzed by CLSM.

### Quantitative Analysis of Intracellular ^•^OH

4T1 cells were seeded in six‐well plates and cultured overnight. After incubation with different treatment components for 12 h, MW‐treated groups were irradiated under microwave (4.95 GHz, 15 W 30 min). Following an additional 12‐h co‐incubation, cells were stained with the ^•^OH fluorescent probe (BBoxiProbe O26) for 30 min, collected, and quantified by FCM.

### Western Blotting Analysis

Equal amounts of protein (measured by BCA kits) were mixed with 5 × SDS loading buffer and boiled at 100  °C for 5 min. After gel electrophoresis and protein transformation, primary antibodies were applied (1:1000) overnight at 4 °C. After washing three times with TBST for 5 min, the membrane was incubated with a Goat anti‐Rabbit/Mouse IgG (H+L)‐HRP conjugate (1: 10 000) for 45 min. Finally, the protein bands were obtained by chemiluminescence.

### Animal Experiments

All animal experiments were conducted in accordance with protocols approved by the Animal Experimental Ethics Committee of Fujian Normal University (Approval No. IACUC‐20230059). Every effort was made to minimize animal suffering and reduce the number of animals used, following the principles of the 3Rs (Replacement, Reduction, and Refinement).

### Hemolysis Assay

Fresh whole‐blood samples were collected from the orbital veins of Balb/c mice. The blood samples were centrifuged at 1000 rpm for 15 min and gently rinsed with PBS to collect erythrocytes. In a typical assay, 500 µL of the diluted erythrocyte suspension was combined with 500 µL Cu_2_O@*E*_G_ suspension at various concentrations. PBS and 3% Triton X‐100 were set as negative and positive controls, respectively. The mixed solutions were then incubated at 37 °C for 2 h and subsequently centrifuged at 10 000 g for 15 min. Optical absorbances of the supernatant at 540 nm were measured by a microplate reader. The hemolysis ratio was calculated according to the following formula:

(1) $$\mathrm{Hemolysis}\;\mathrm{ratio}\;\;\left(\%\right)=\frac{A_{sample}-A_{saline}}{A_{water}-A_{saline}}\;\times 100\%$$

### In Vivo Biodistribution

4T1 cells (1 × 10^6^ cells per mouse) were subcutaneously injected into BALB/c to establish a breast tumor‐bearing mouse model. The mice were subsequently divided into three groups for active targeting assessment. Upon reaching a tumor volume of ≈150 mm^3^, ICG, ICG‐labeled *E. coli*, and Cu_2_O@*E*_G_ (at a concentration of ≈1 × 10^7^ CFU per mouse) were intravenously administered to the mice. In vivo fluorescence imaging of the mice was conducted at 0.5, 2, 4, 8, 12, and 24 h using IVIS Spectrum imaging. After 24 h, the mice were euthanized under anesthesia, and both tumors and major organs (including the heart, liver, spleen, lung, and kidney) were collected for *ex vivo* imaging. Simultaneously, the fluorescence intensity in each tissue was measured.

To study the in vivo biodistribution of bacteria, the liver, spleen, lung, heart, kidney, and tumor were collected and weighted at 24 h, 7 days, and 18 days after administering Cu_2_O@*E*_G_ (≈1 × 10^7^ CFU per mouse) through the tail vein. The collected tissues were sieved by grinding through a cell sieve to obtain a tissue suspension. Then, the 200 µL of each dilution was spread onto LB agar plates with ampicillin before overnight incubation at 37 °C. Colonies on the plates were counted and photographed.

### In Vitro ICG Release Kinetics

The ICG‐loaded bacteria were resuspended in PBS buffer (pH 6.5) to simulate tumor microenvironmental conditions. Samples were incubated at 37 °C with gentle shaking, with aliquots centrifuged at designated time points to collect supernatants for quantification of ICG fluorescence intensity using UV–vis spectrophotometry.

### Pharmacokinetic Analysis

Cu_2_O@*E*_G_ biorobot was intravenously administered to mice via the tail vein. Blood samples (10 µL) were collected from the tail vein at designated time points, followed by harvesting major organs (liver, kidneys, heart, and spleen) and the tumor at 24 h and 18 days post‐injection. Copper ion concentrations in both blood and tissues were quantified using AAS.

### In Vivo Antitumor Therapy

To evaluate antitumor efficacy, 1 × 10^6^ 4T1 cells were subcutaneously injected into Balb/C mice (female, 6 weeks). When the tumors reached ≈100 mm^3^, mice were randomly divided into ten groups (for each group, *n* = 6). Each group was treated with various formulations (PBS, Cu_2_O, *E*_G_, Cu_2_O@*E*, Cu_2_O@*E*_G_) via tail vein injection. Especially, the group that required microwave therapy was exposed to 30 min of localized microwave radiation on days 3 and 7 post‐injection. During the treatment period, the tumor volumes and body weight were recorded every 2 days until day 15. After 14 day‐therapy, the mice in diverse groups were sacrificed. Along with, tumors were collected, weighted, and photographed. Besides, the major organs (including heart, liver, spleen, lung, and kidney) and tumors were harvested to be stained with H&E for histology studies. Meanwhile, tumors were fixed with 4% PFA and sectioned for further H&E staining and immunohistochemistry.

The tumor volume was calculated using the formula:

(2) $$V=\frac{a\times b^{2}}{2}$$

where a refers to tumor length and b refers to tumor width, supposing a > b.

### Flow Cytometry of TILs

For analysis of immune cells in tumors and lymph nodes, on day 18 after the treatment, the tumors and lymph nodes were collected and disaggregated in cold PBS solution containing 1% FBS (TPBS) buffer. Then, the sieved tissue was ground with a separate cell sieve to obtain a single cell suspension. The collected tumor tissue single cell suspensions were treated with RBC lysis buffer for 5 min and washed with TPBS buffer. Then the collected cells were isolated from immune infiltrating lymphocytes (TILs) by Percoll. After that, the obtained TILs were stained with fluorescence‐labeled antibodies and then measured using flow cytometry.

### Cytokine Detection

Balb/C mice bearing 4T1 tumors were subject to different treatments. Blood was collected, and the contents of cytokines (IFN‐γ and TGF‐β) in plasma were quantified using corresponding ELISA kits according to the manufacturer's instructions.

### Biosecurity Estimations

Six healthy Balb/C mice were intravenously administered with PBS, Cu_2_O@*E* and Cu_2_O@*E*_G_ at a dose of 1 × 10^7^ CFU per mouse used for biosafety assessment. The weight of the mice was recorded every five days, and blood samples were collected at 60 days for hematological analysis and serum biochemistry measurements. Additionally, the major organs, including the heart, liver, spleen, lungs, and kidneys, were harvested and weighed. Besides, these organs were stained with H&E for histological examination.

### Statistical Analysis

All quantitative data are presented as mean ± standard deviation (SD). Prior to statistical analysis, outliers beyond ± 3SD were excluded to ensure data normality. For in vivo experiments, a sample size of *n* = 5 per group was used, while in vitro studies employed *n* = 3 biological replicates per condition, as specified in the respective figure legends. Statistics were analyzed by one‐way or two‐way analysis of variance (ANOVA), followed by two‐sided Student's *t*‐test using GraphPad Prism 8.0.2. Differences were considered significant when the *p*‐value was less than or equal to 0.05. * *p* < 0.05, ** *p* < 0.01, *** *p* < 0.001, and **** *p* < 0.0001. ns is not significant.

## Conflict of Interest

The authors declare no conflict of interest.

## Supporting information



Supporting Information

## Data Availability

The data that support the findings of this study are available from the corresponding author upon reasonable request.;
