# Correction to “Microwave‐Activated Bacterial Biorobot for Multimodal Cancer Therapy”

**DOI:** 10.1002/advs.202520958

**Published:** 2025-12-21

H. Zhuang, Y. Zhang, Y. Fu, D. Ke, Q. Chen, S. Shao, P. Xue, Y. Chen, X. Zeng, S. Yan, Microwave‐Activated Bacterial Biorobot for Multimodal Cancer Therapy. *Adv. Sci*. **2025**, 12, e04603. https://doi.org/10.1002/advs.202504603

In the published article, we found that the FDX1‐staining image of group 6 (PBS + MW) in Figure 6g was improperly used during organizing the figures. The corrected figures are shown below.

Corrected Figure 6g:


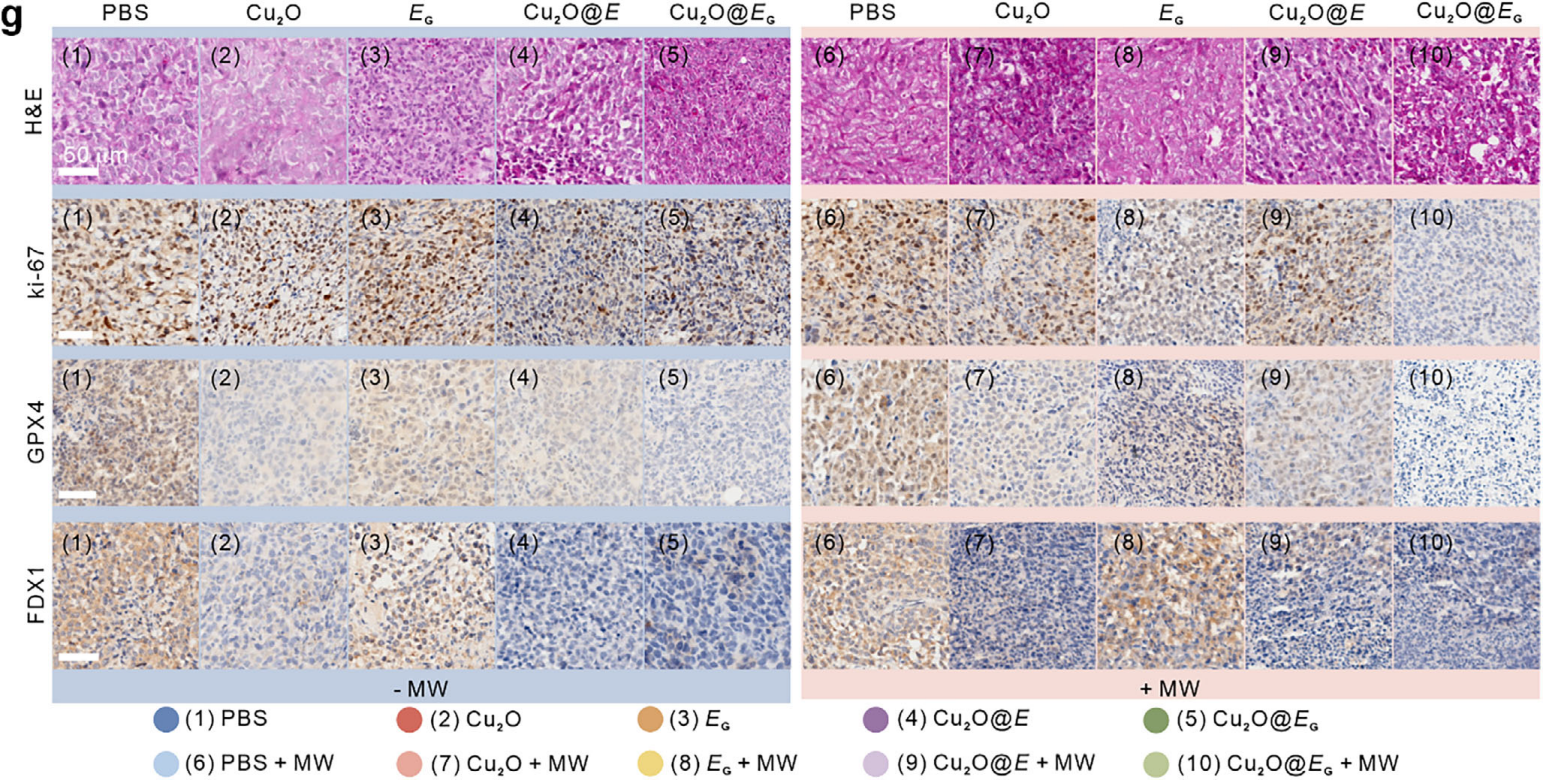


In Figure S38, the liver and kidney images for the “PBS + MW” group were inadvertently duplicated from the “Cu_2_O@*E*_G_ ‐ MW” group during the layout of the images. The corrected Figure S38 is presented below.

Corrected Figure S38 (Supporting Information):


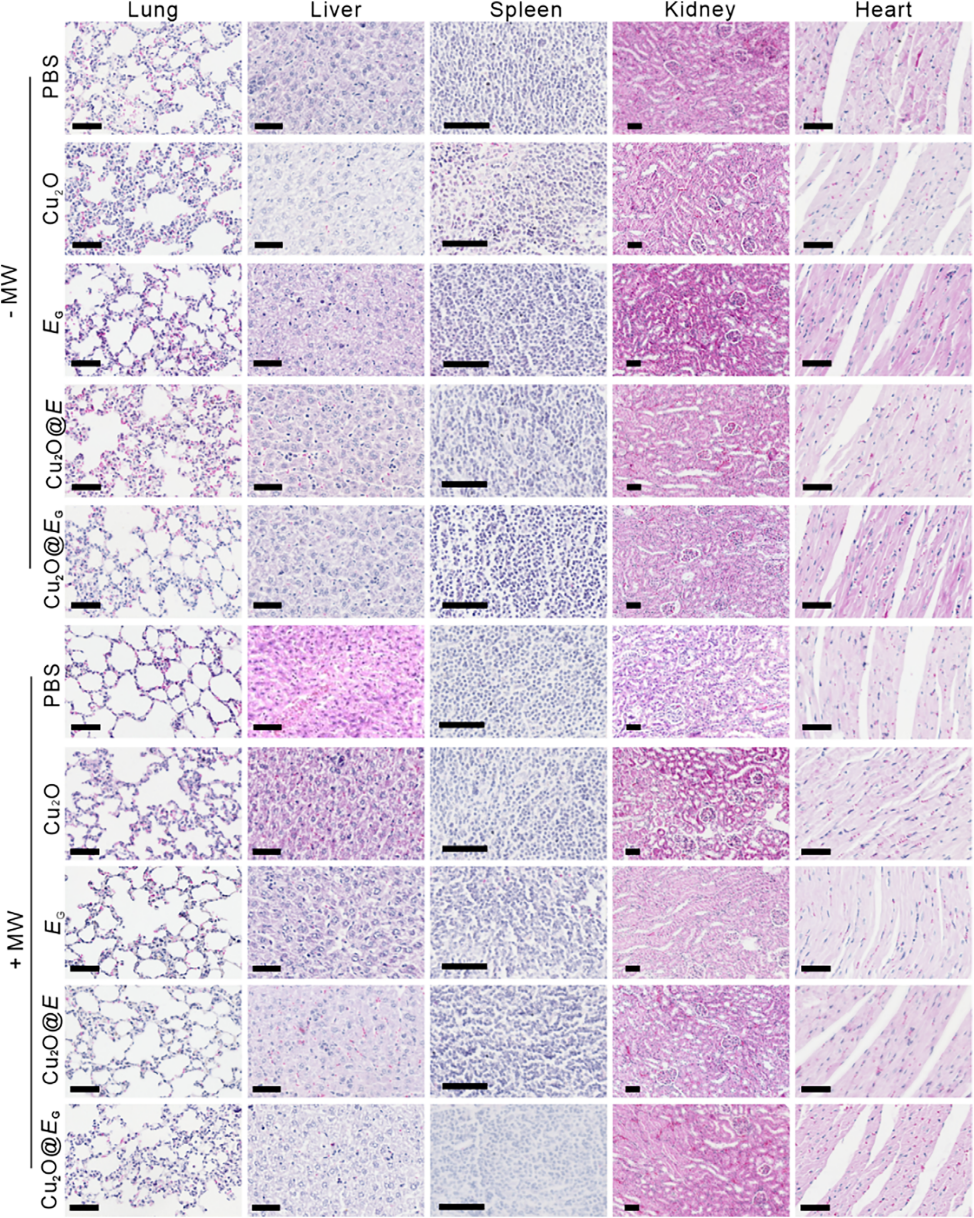


These corrections do not affect the overall findings and conclusions of the paper.

We sincerely apologize for these errors.

