# Supplementary material for: Microwave‐Activated Bacterial Biorobot for Multimodal Cancer Therapy
Source: Adv Sci (Weinh). 2025 Aug 11;12(41):e04603. doi: 10.1002/advs.202504603 (PMC12591154; doi:10.1002/advs.202504603)
Supplement: Supplementary file 1 — Supporting Information [file ADVS-12-e04603-s001.docx]

Supporting Information

Microwave-Activated Bacterial Biorobot for Multi-Modal Cancer Therapy

*Huilan Zhuang, Yongjian Zhang, Yajuan Fu*, Dangjin Ke, Qi Chen*, Sijie Shao, Panpan Xue, Yuanchun Chen, Xuemei Zeng*, Shuangqian Yan**

H. L. Zhuang, S. J. Shao, P. P. Xue, Y. C. Chen, X. M. Zeng, S. Q. Yan

Fujian Provincial Key Laboratory of Flexible Electronics, Strait Institute of Flexible Electronics (SIFE Future Technologies) Fujian Normal University Fuzhou 350007, P. R. China

E-mail: xmzeng@fjnu.edu.cn; [ifeshqyan@fjnu.edu.cn](mailto:ifeshqyan@fjnu.edu.cn)

Y. J. Zhang

The Sixth Affiliated Hospital of Harbin Medical University, Harbin, Heilongjiang, 150000, China.

Y. J. Fu, D. J. Ke, Q. Chen

Key Laboratory of Innate Immune Biology of Fujian Province, Biomedical Research Center of South China, College of Life Sciences, Fujian Normal University, 1 Keji Road, Fuzhou 350117, P. R. China

E-mail: fuyajuan@fjnu.edu.cn; [chenqi@fjnu.edu.cn](mailto:chenqi@fjnu.edu.cn)


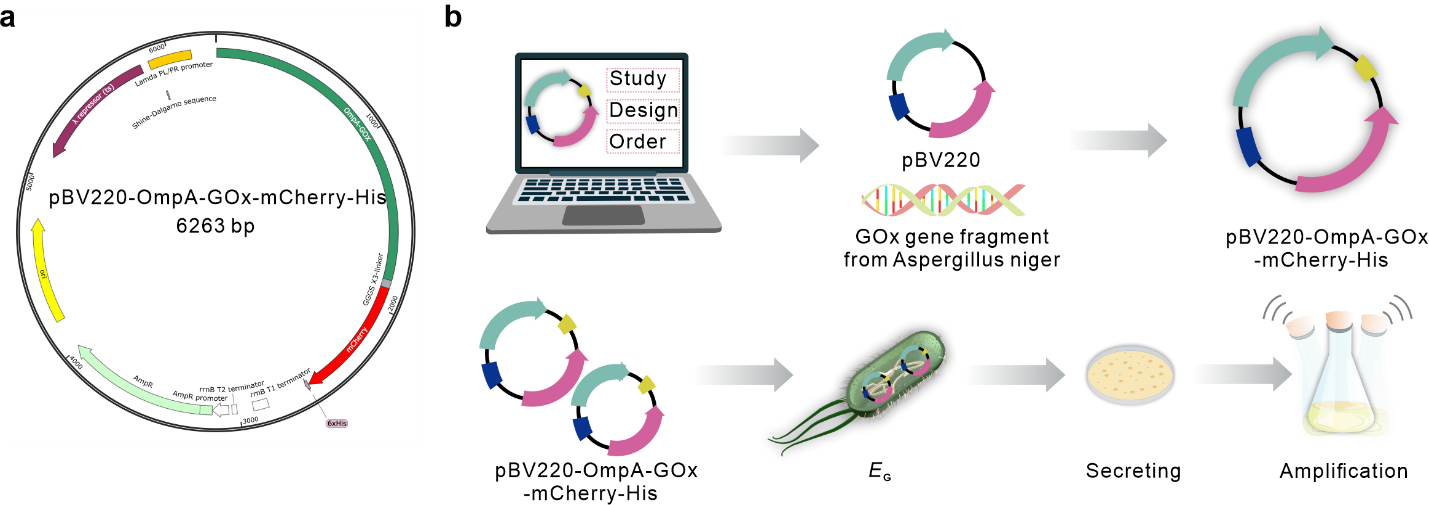


Figure S1. Construction and preparation of engineered bacteria. a) pBV220-GOx-mcherry plasmid map. b) Schematic illustration of *E*_G_ preparation.


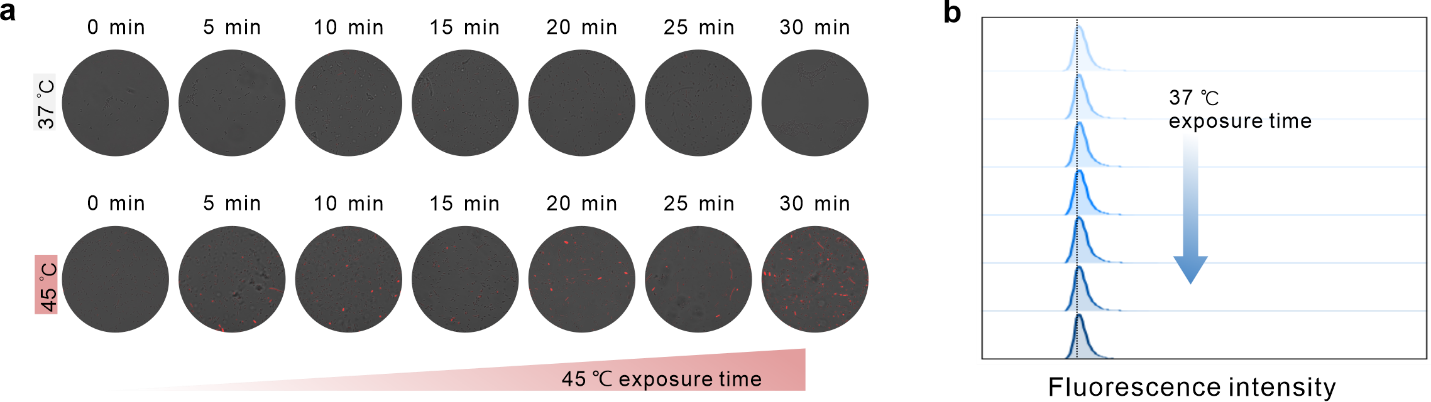


Figure S2. *In vitro* response expression validation. a) Hypothermal-mediated and illumination time-dependence of *E*_G_ studied by CLSM. b) Fluorescence semi-quantification of *E*_G_ expressed mCherry by FCM.


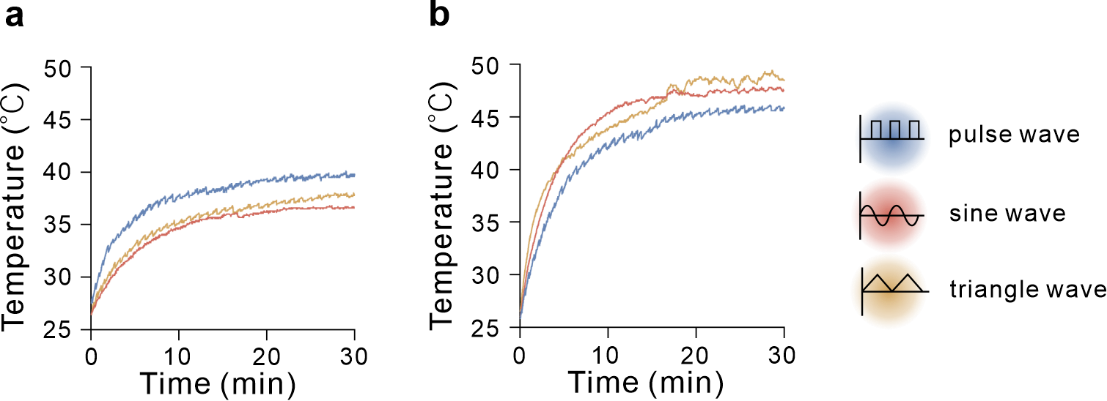


Figure S3. Warming curves of PBS under continuous irradiation of microwaves of different waveforms at 10 W a) and 20 W b) irradiation of different waveforms.


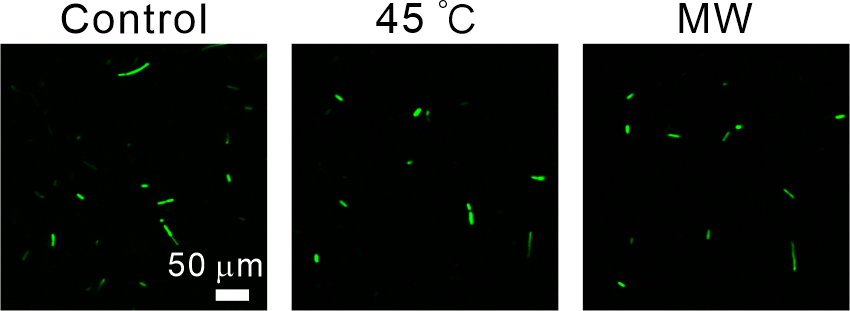


**Figure S4.** FDA staining of *E*_G_ treated by different formulations.


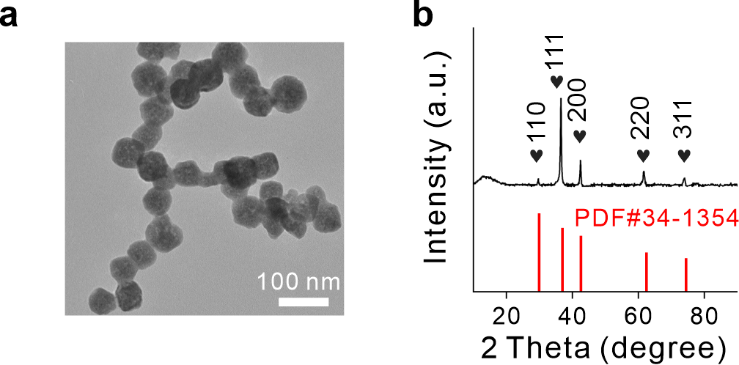


Figure S5. Characterization of Cu_2_O nanoparticles. a) TEM image of the synthesised Cu_2_O nanoparticles. b) XRD analysis of the synthesised Cu_2_O nanoparticles.


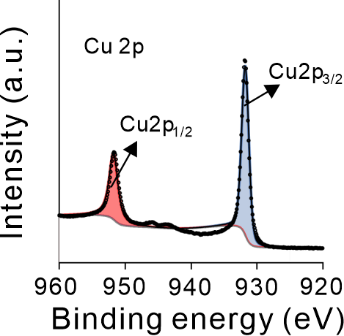


Figure S6. Cu 2p XPS spectra of the synthesised Cu_2_O nanoparticles.


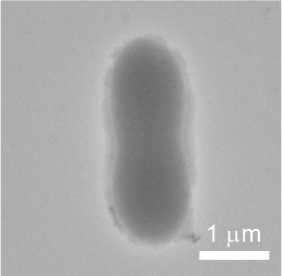


Figure S7. TEM image of the pure *E*_G_.


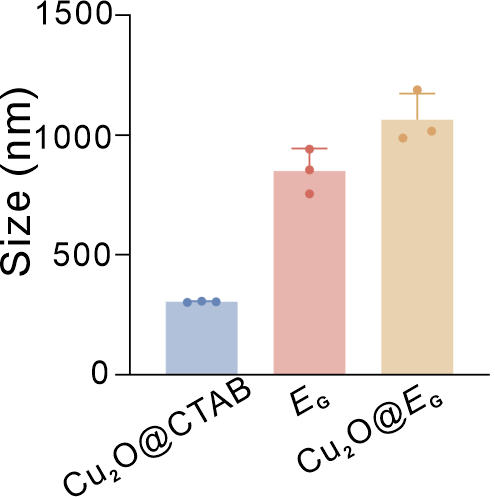


Figure S8. The DLS analysis of Cu_2_O@CTAB, *E*_G_ and Cu_2_O@*E*_G_. (n = 3 samples). Data are presented as mean ± S.D.


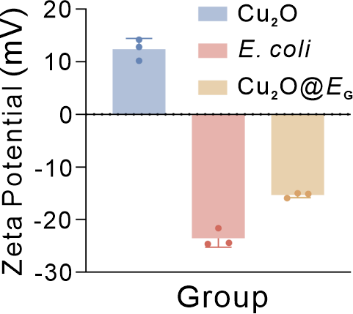


Figure S9. Zeta potentials of t Cu_2_O, *E. coli* and Cu_2_O@*E*_G_. (n = 3 samples). Data are presented as mean ± S.D.


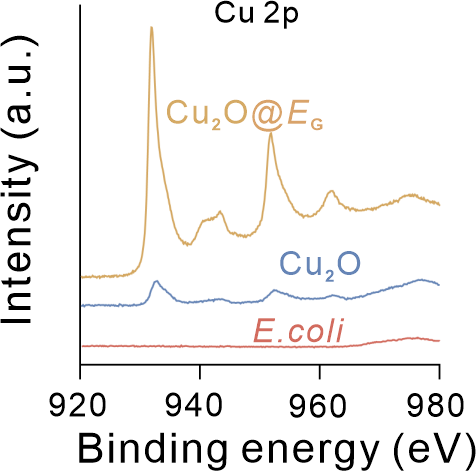


Figure S10. Cu 2p XPS spectra of Cu_2_O, *E. coli* and Cu_2_O@*E*_G_.


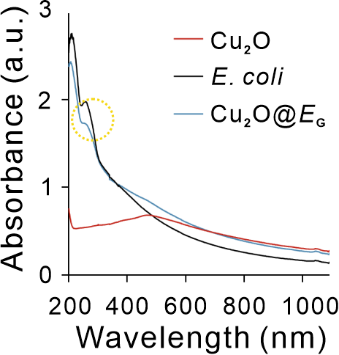


Figure S11. UV-vis absorption spectrum of Cu_2_O, *E. coli* and Cu_2_O@*E*_G_.


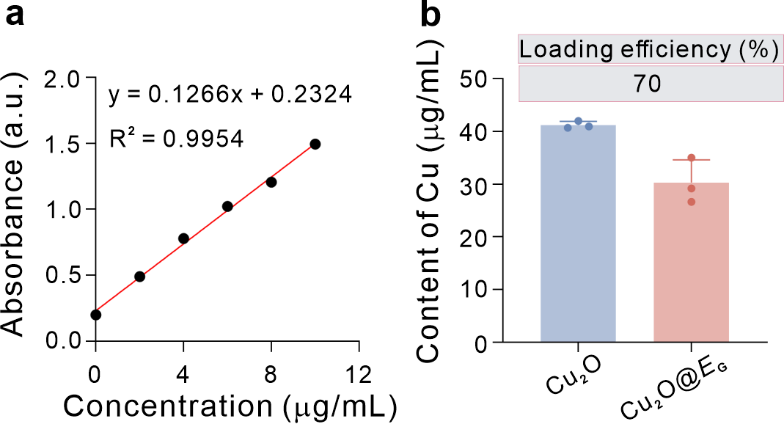


**Figure S12.** Computational analysis of Cu_2_O loading rate. a) Standard curve for copper content by AAS. b) The Cu content before and after Cu_2_O loading was obtained by AAS analysis (n = 3 samples). Data are presented as mean ± S.D.


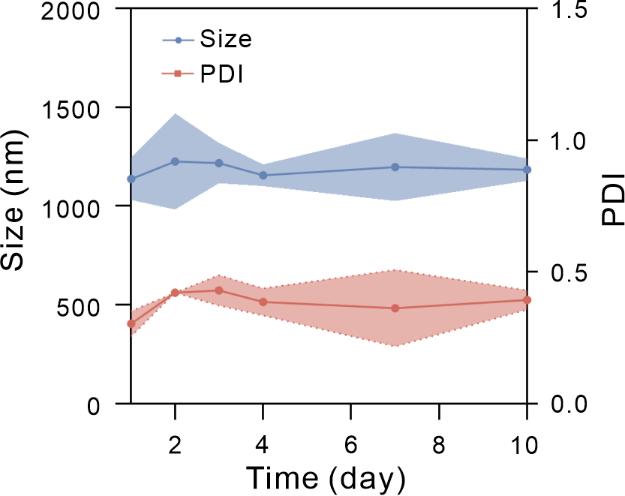


**Figure S13.** Changes in hydrodynamic diameter of Cu_2_O@*E*_G_ in 1% FBS-supplemented medium over a 10-day period (n = 3). Data are presented as mean ± S.D.


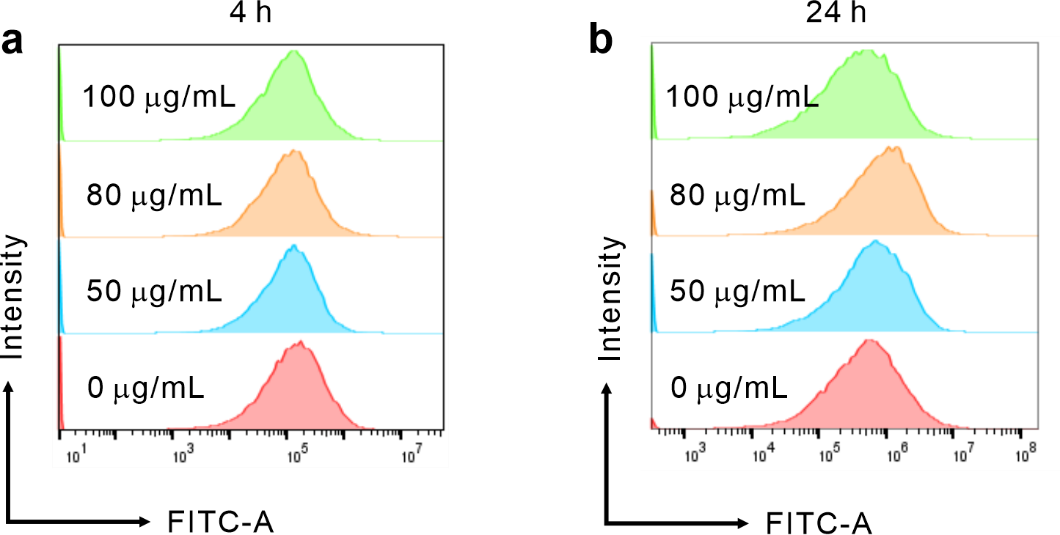


**Figure S14.** Effects of varying Cu_2_O NPs concentrations and incubation durations on *E*_G_ viability assessed *via* FDA staining.


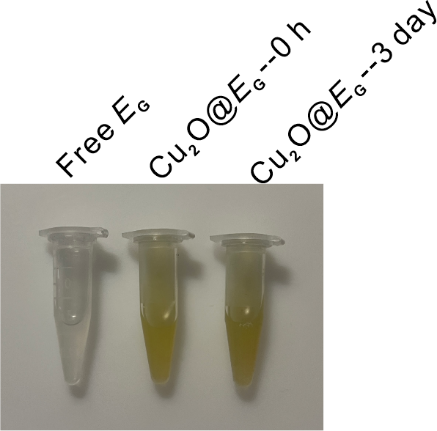


**Figure S15.** Photos of the solution at different times.


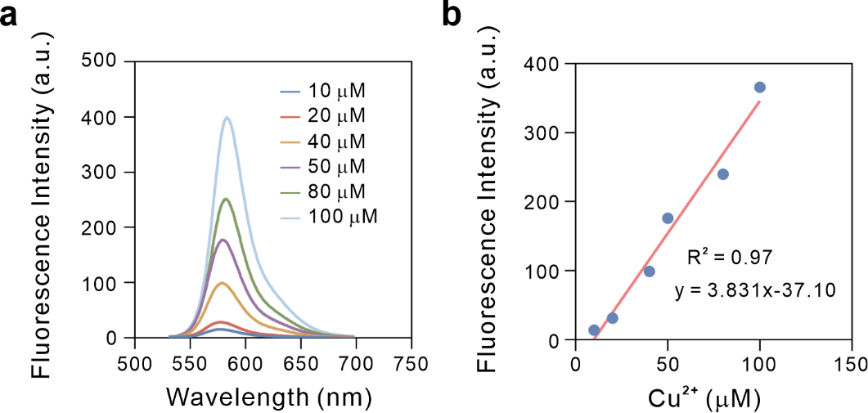


**Figure S16.** Calibration curve of RBH for Cu^2+^ detection. a) Fluorescence spectra of RBH in the presence of varying Cu^2+^ concentrations (10-100 μM). b) Linear relationship between fluorescence intensity and Cu^2+^ concentration.


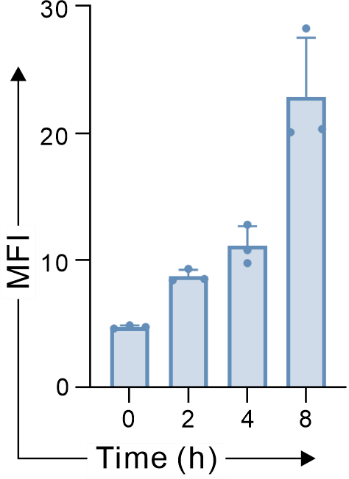


**Figure S17.** The corresponding mean fluorescence intensity (MFI) quantifications of Cu ions with different treatment time. Data are presented as mean ± S.D. (n = 3 samples).


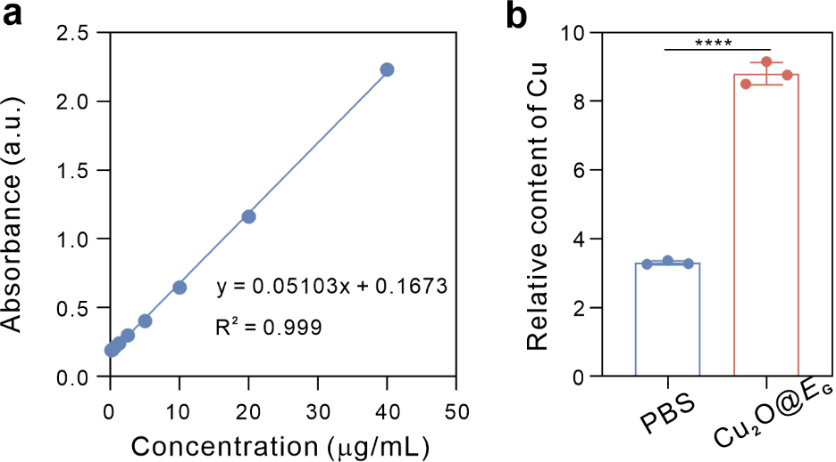


**Figure S18.** Cellular copper ion concentration detection. a) Standard curve for copper ion quantification. b) Copper content in 4T1 cells treat with PBS or 6 h Cu_2_O@*E*_G_. Data are presented as mean ± S.D. (n = 3 samples). Statistical analysis was calculated by using one-way analysis of variance with a Tukey’s test (****P < 0.0001).


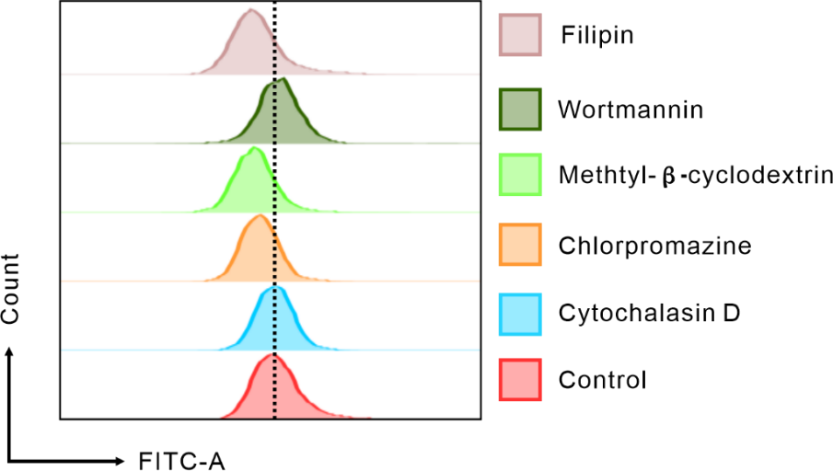


**Figure S19.** Differential effects of endocytic pathway inhibitors on Cu_2_O@*E*_G_ uptake in 4T1 cells.


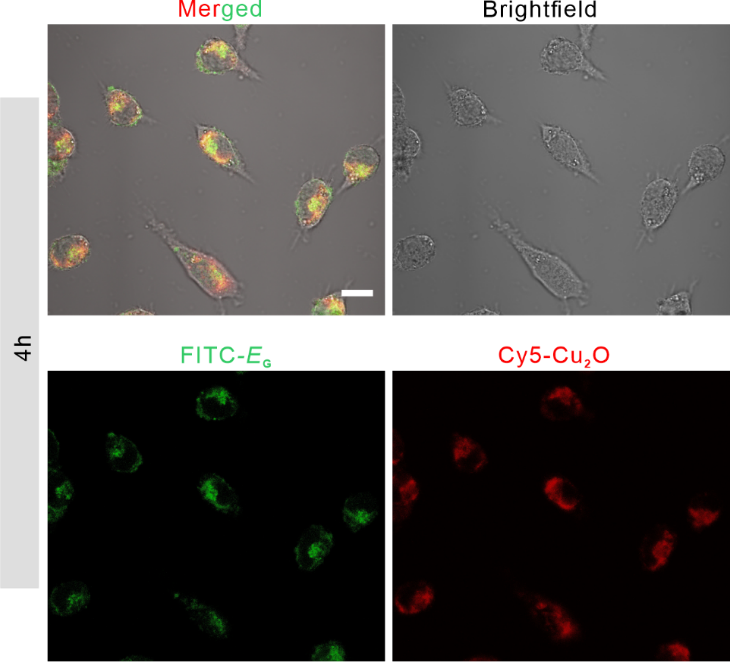


**Figure S20.** Representative images from CLSM imaging of intracellular localization of Cy5-Cu_2_O and FITC-*E*_G_. Scale bars: 20 μm.


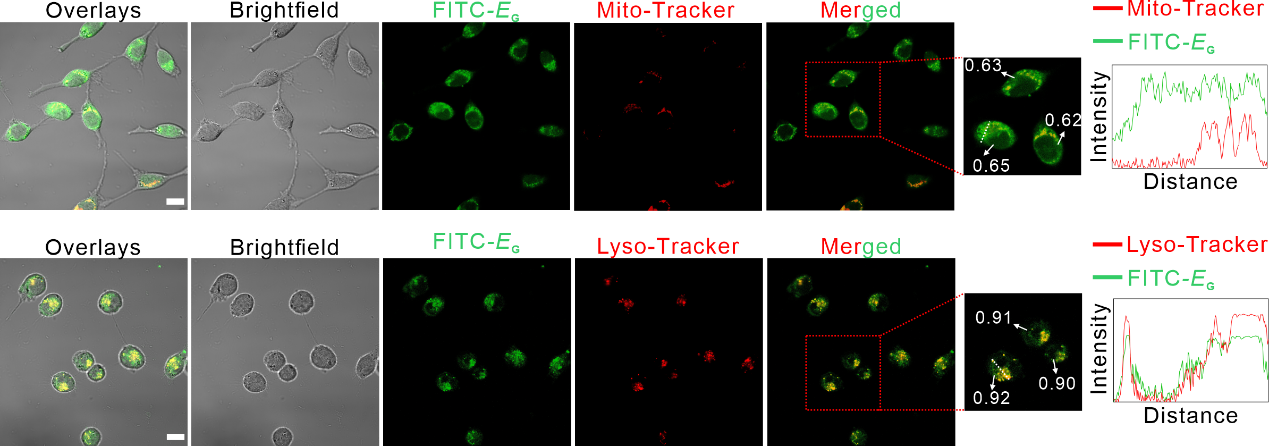


**Figure S21.** Representative CLSM images and plot profiles of colocalization between FITC-labeled Cu_2_O@*E*_G_ (green) and LysoTracker (red) or MitoTracker Deep Red (red) in 4T1 cells. Scale bars, 20 μm.


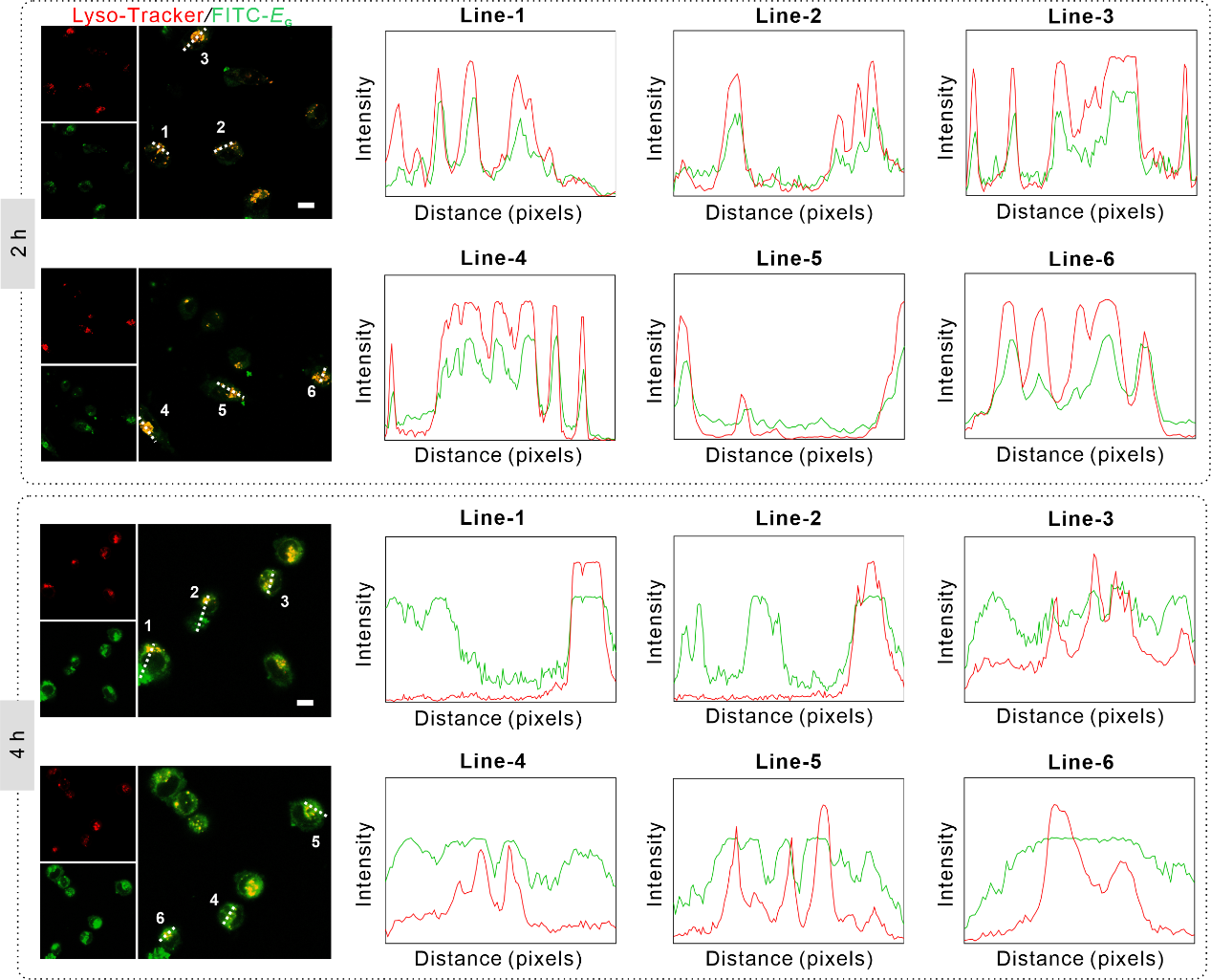


**Figure S22.** Colocalization of FITC-labeled Cu_2_O@*E*_G_ (green) and lysosomes (LysoTracker Red) in 4T1 cells at different time points. Scale bars: 20 μm.


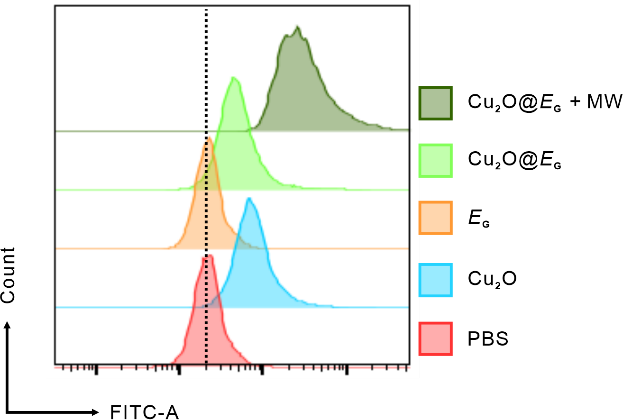


**Figure S23.** FCM results of cellular ^•^OH content in 4T1 cells after different treatments.


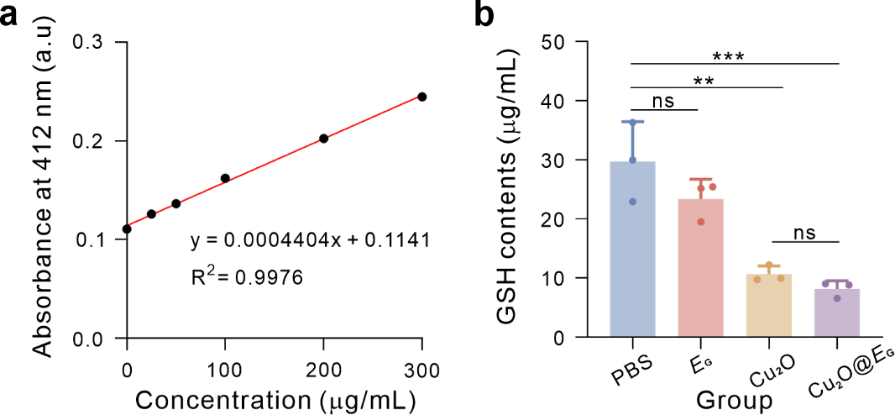


Figure S24. Detection of intracellular GSH content after different treatments. a) Standard curve of GSH content measured by DTNB kit. b) Intracellular GSH content after different treatments. Data are presented as mean ± S.D. (n = 3 samples). Statistical analysis was calculated by using one-way analysis of variance with a Tukey’s test (***P < 0.001; **P < 0.01 and ns > 0.05).


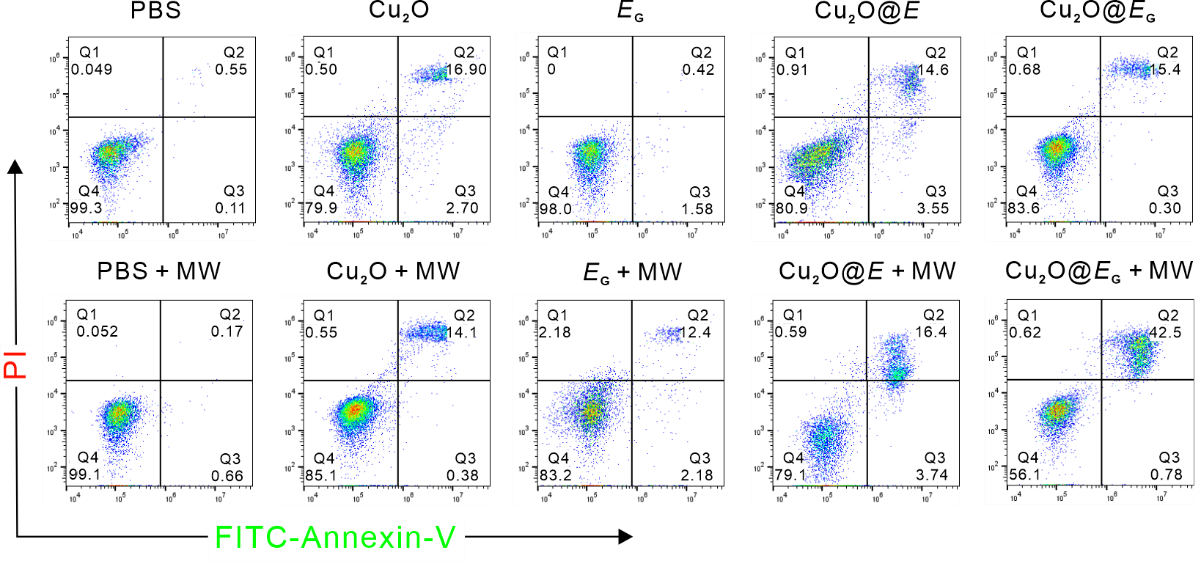


**Figure S25.** Apoptosis study of 4T1 cells with different treatments by FCM.


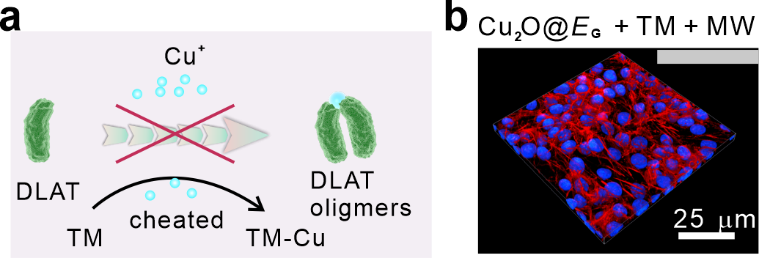


Figure S26. Assessment of DLAT oligomerization in 4T1 cells. a) Schematic representation of the process of oligomerization of DLAT proteins with Cu^+^ to form oligomerized DLAT and the organization of this process by TM through chelation of Cu^+^. b) TM blocked DLAT oligomerization which was visually presented by immunofluorescence staining.


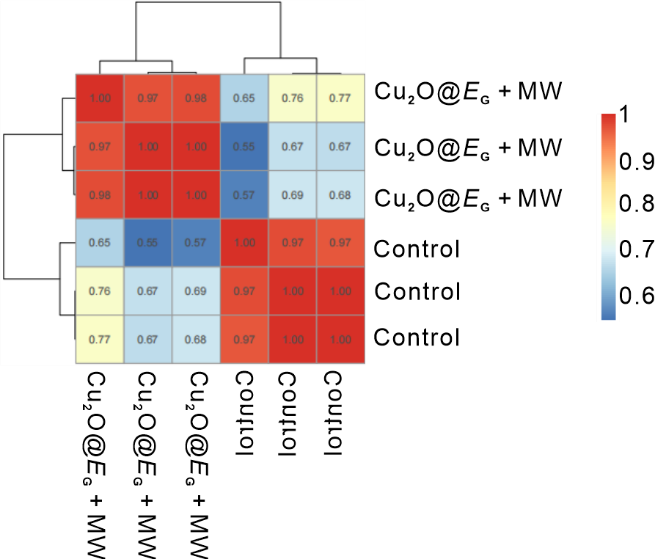


Figure S27. Heat map illustrates the correlation between samples. (n = 3 samples).


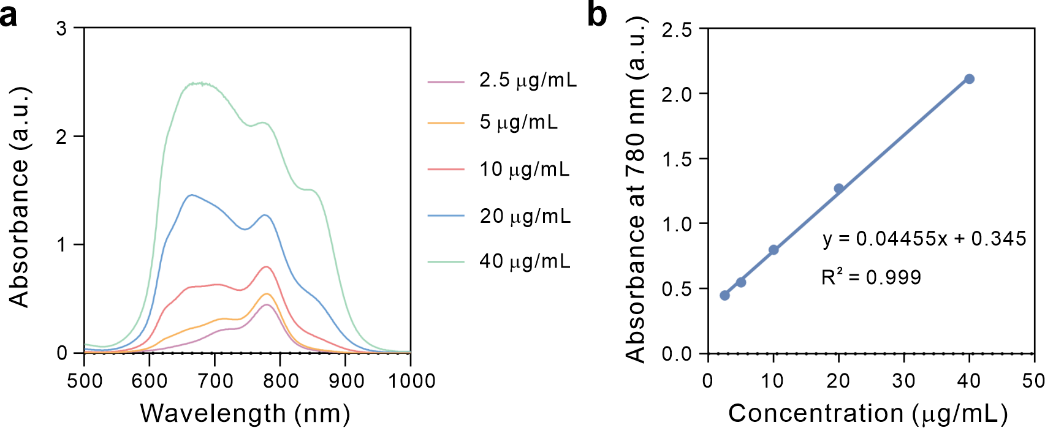


**Figure S28.** a) Absorption spectra of ICG at varying concentrations. b) Linear calibration curve at 780 nm.


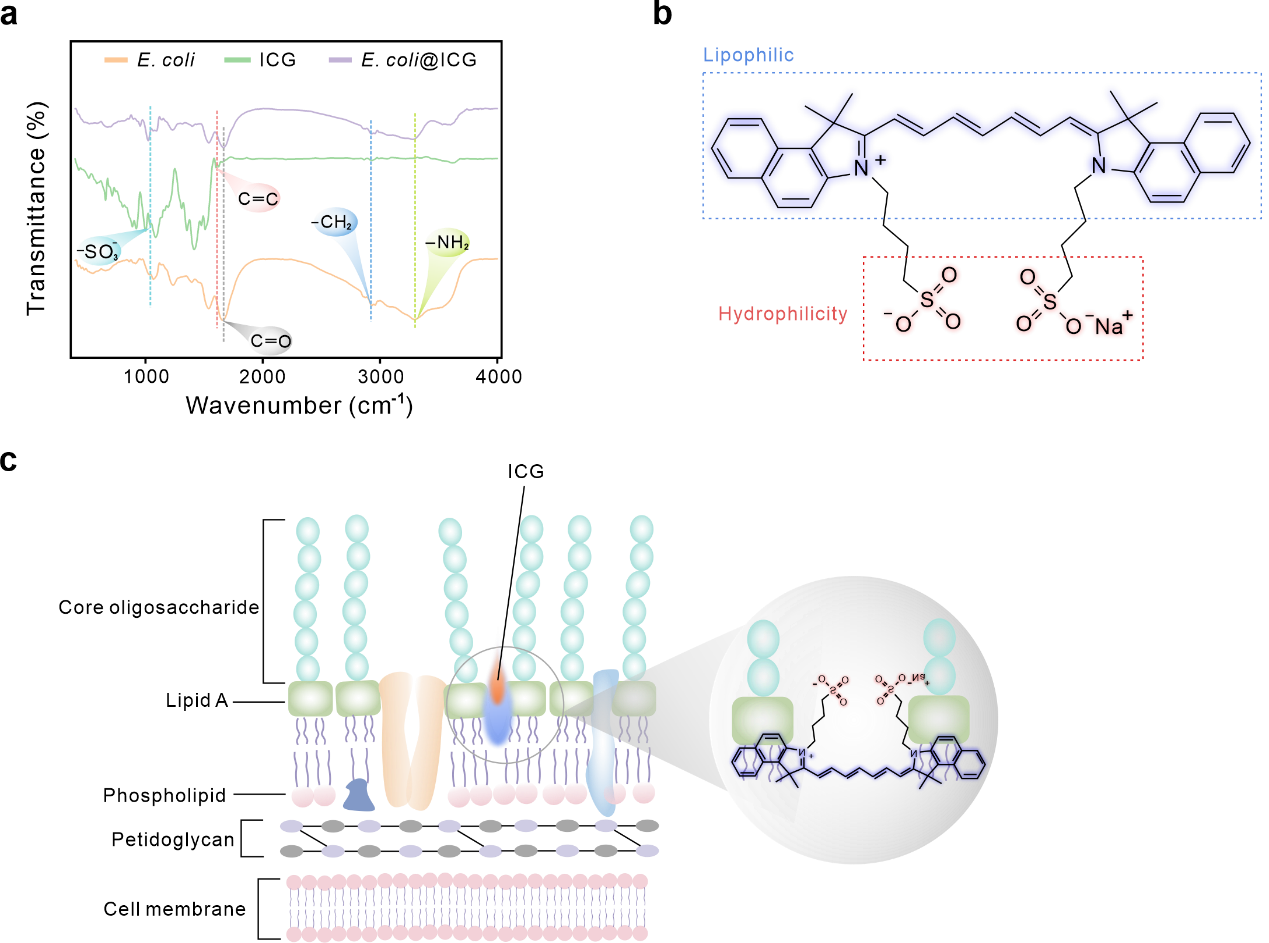


**Figure S29.** a) Fourier-transform infrared (FTIR) spectroscopic analysis of *E. coli*, ICG and *E. coli*@ICG. b) Chemical structure of ICG. c) Schematic illustration of bacterial membrane structure and ICG loading mechanism on the bacterial surface.


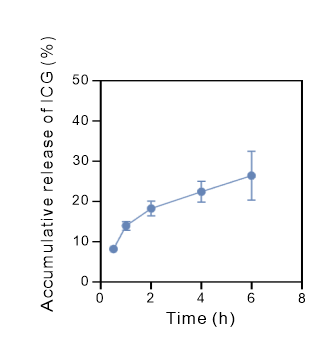


**Figure S30.** Time-dependent cumulative release profile of ICG in PBS (pH 6.5) at 37°C. Data are presented as mean ± S.D. (n = 3 independent experiments).


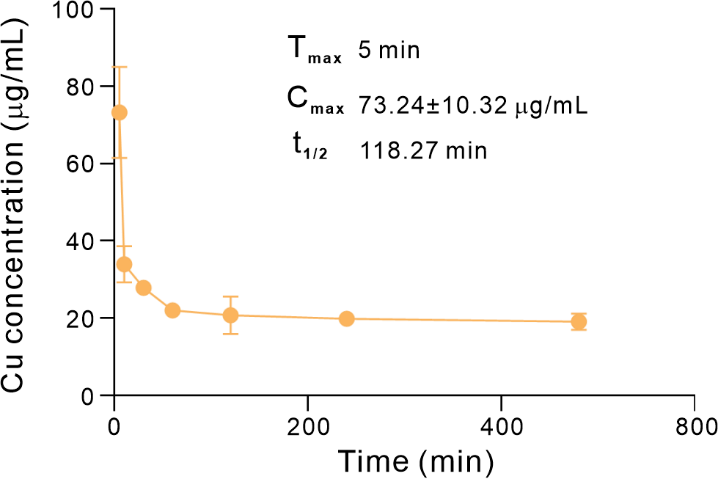


**Figure S31.** Pharmacokinetic analysis of Cu_2_O@*E*_G_. C_max_: peak concentration; T_max_: time to peak concentration; t_1/2_: half-life. Data are presented as mean ± S.D. (n = 3 independent experiments).


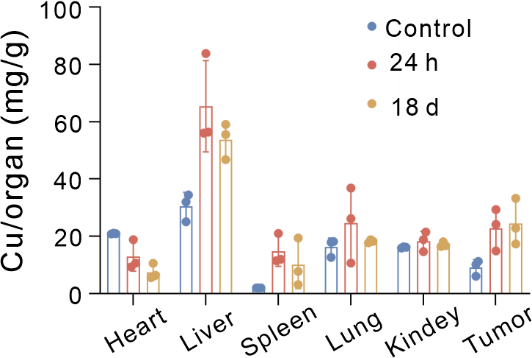


**Figure S32.** Cu content in main organs under different treatment. Data are presented as mean ± S.D. (n = 3 independent experiments).


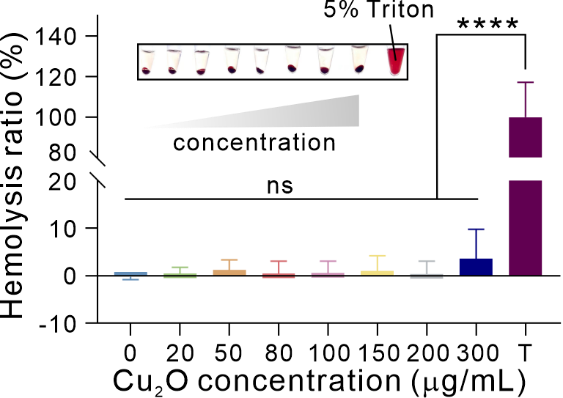


Figure S33. Hemolytic analysis of *E*_G_ loaded with different concentrations of Cu_2_O. Data are presented as mean ± S.D. (n = 3 samples). Statistical analysis was calculated by using one-way analysis of variance with a Tukey’s test (****P < 0.0001 and ns > 0.05).


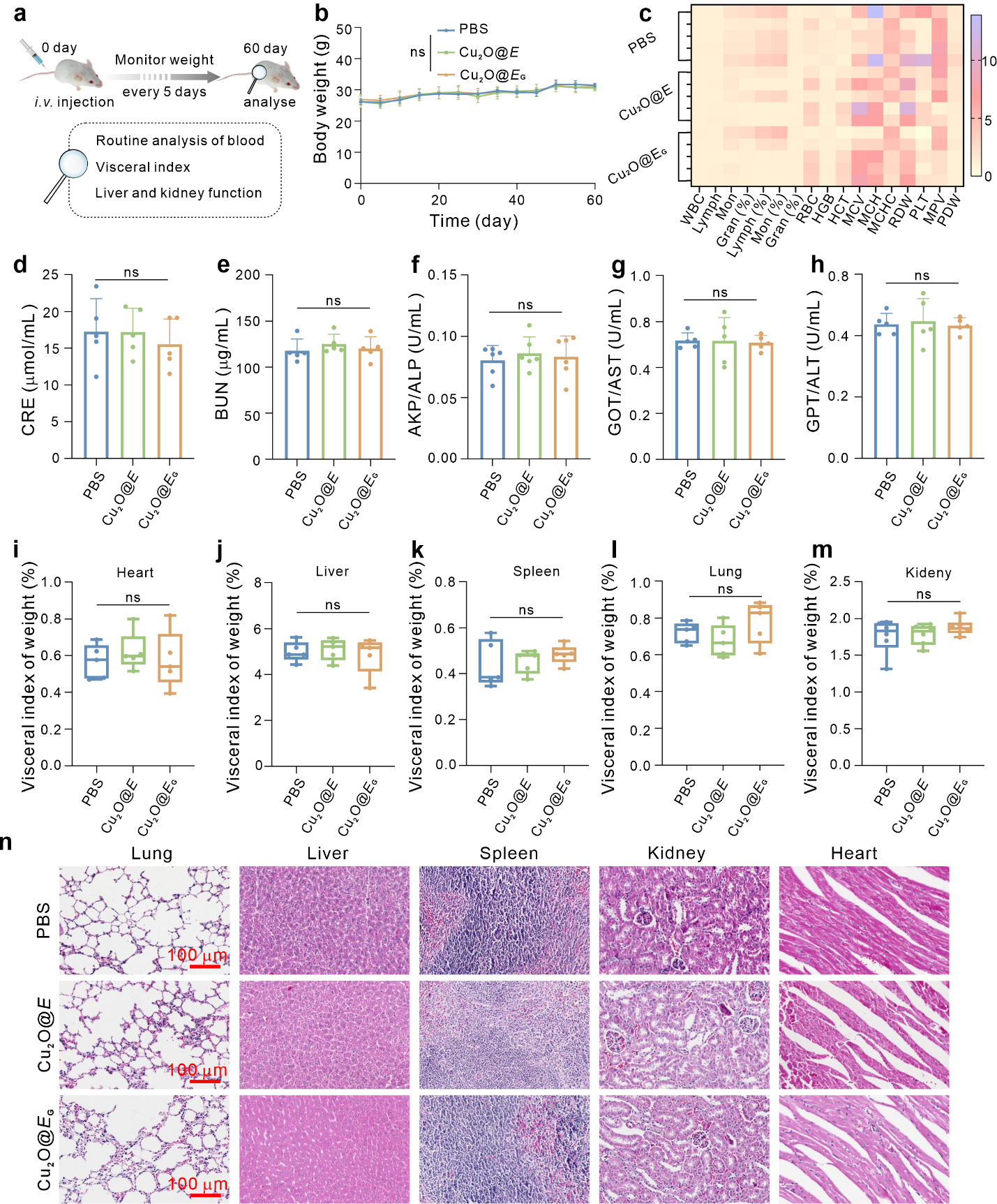


Figure S34. Biosafety evaluation of Cu_2_O@*E*_G_. a) Schematic diagram of the experimental cycle for long-term toxicity. b) Body weight change curves of mice in different groups during the 60-day. c) Blood biochemistry analysis and complete blood count of mice in different groups. white blood cells (WBC), red blood cells (RBC), hemoglobin (HGB), hematocrit (HCT), mean corpuscular hemoglobin (MCH), mean corpuscular hemoglobin concentration (MCHC), mean corpuscular volume (MCV), and platelets (PLT). d-h) Serum biochemistry analysis of mice. d) creatinine (CRE), e) blood urea nitrogen (BUN), f) alkaline phosphatase (AKP/ALP) alanine aminotransferase (ALT), and g) aspartate aminotransferase (AST). All data are presented as mean ± S.D. (n = 4 mice). i-m) Visceral index of mice weight. n) H&E staining images of histological sections in heart, liver, spleen, lung, and kidney after different treatments for 60 days. Statistical analysis was calculated by using one-way analysis of variance with a Tukey’s test (ns > 0.05).


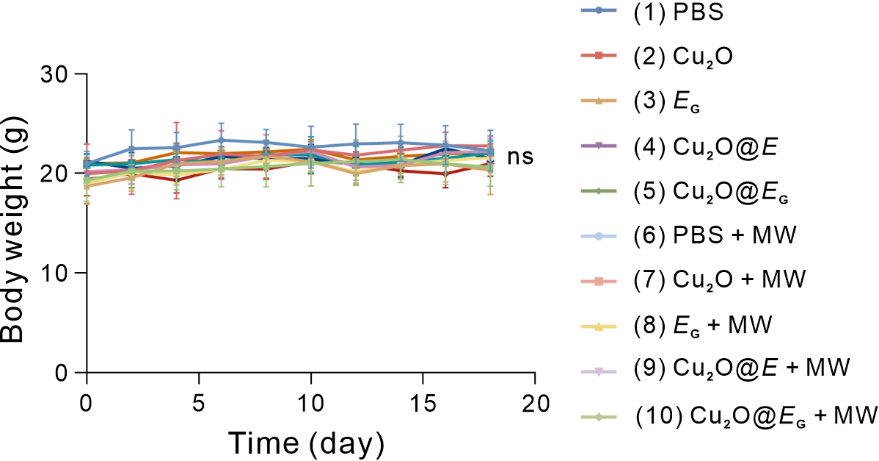


Figure S35. Body weight change curves of mice in different groups during the 18-day treatment. Data are presented as mean ± S.D. (n = 5 mice). Statistical analysis was calculated by using one-way analysis of variance with a Tukey’s test (ns > 0.05).


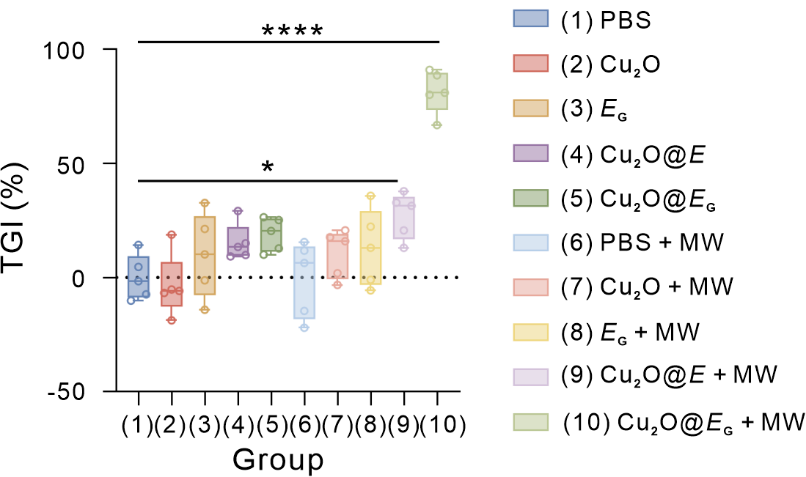


Figure S36. Tumor inhibition rate in mice in different treatment groups. Data are presented as mean ± S.D. (n = 5 mice). Statistical analysis was calculated by using one-way analysis of variance with a Tukey’s test (****P < 0.0001 and *P < 0.1).


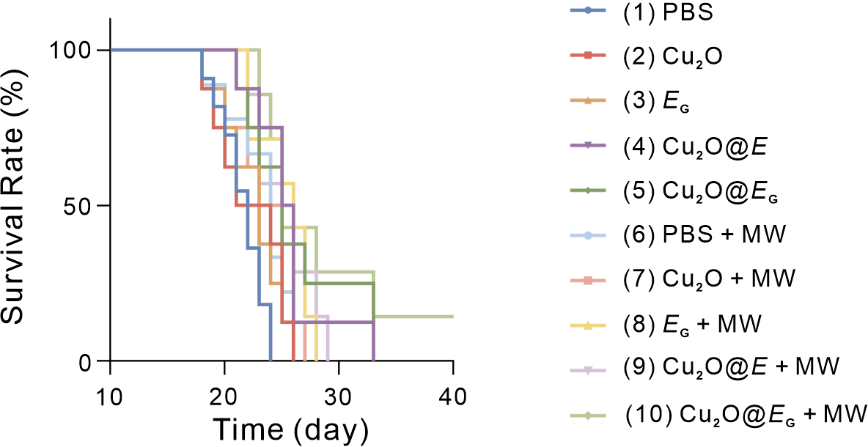


Figure S37. Survival curves for different treatment groups upon B16-F10 tumor-bearing mouse models. (n = 6 mice).


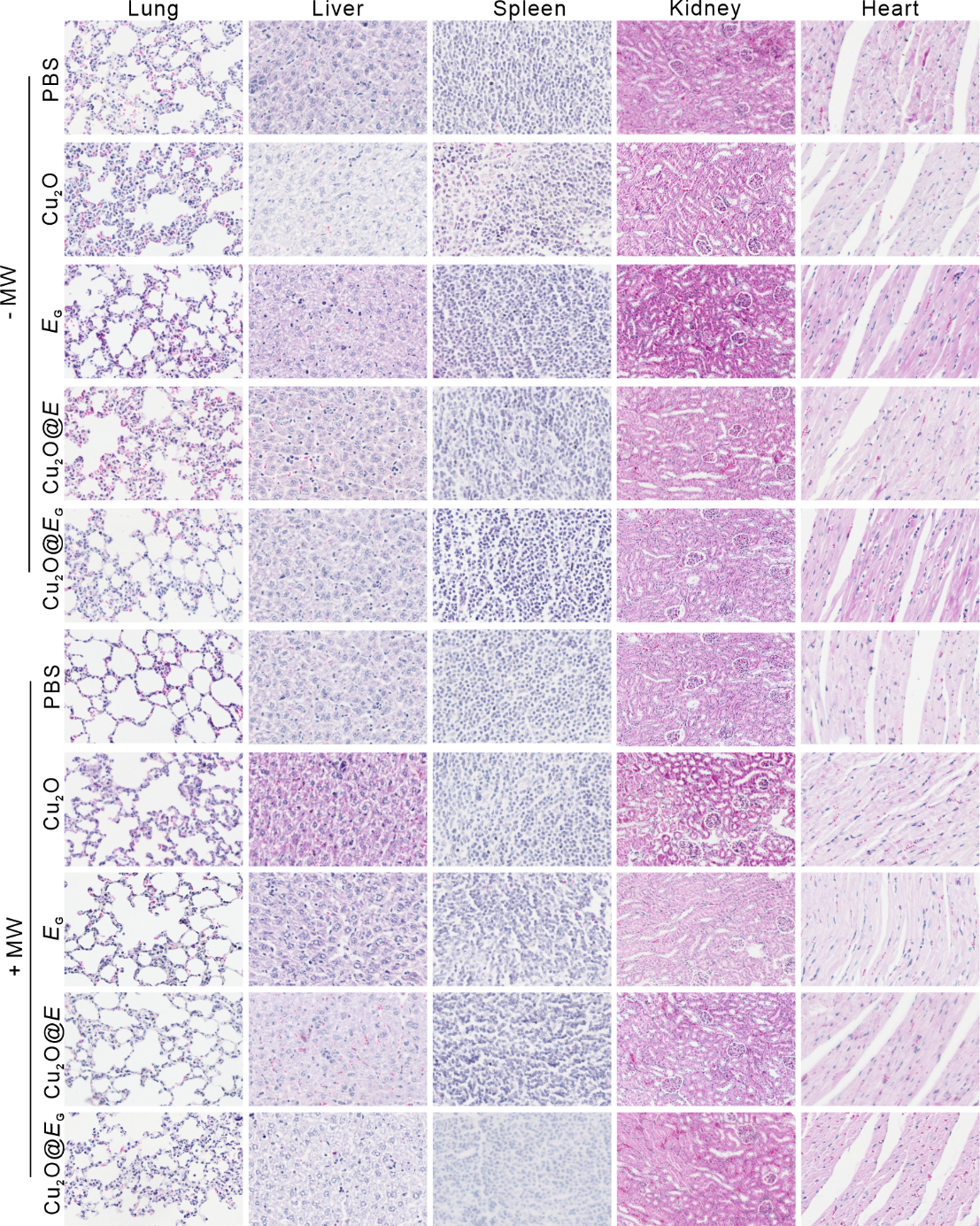


Figure S38. H&E histological analyses in major organs (heart, liver, spleen, lung and kidney) after 18 days treatment.


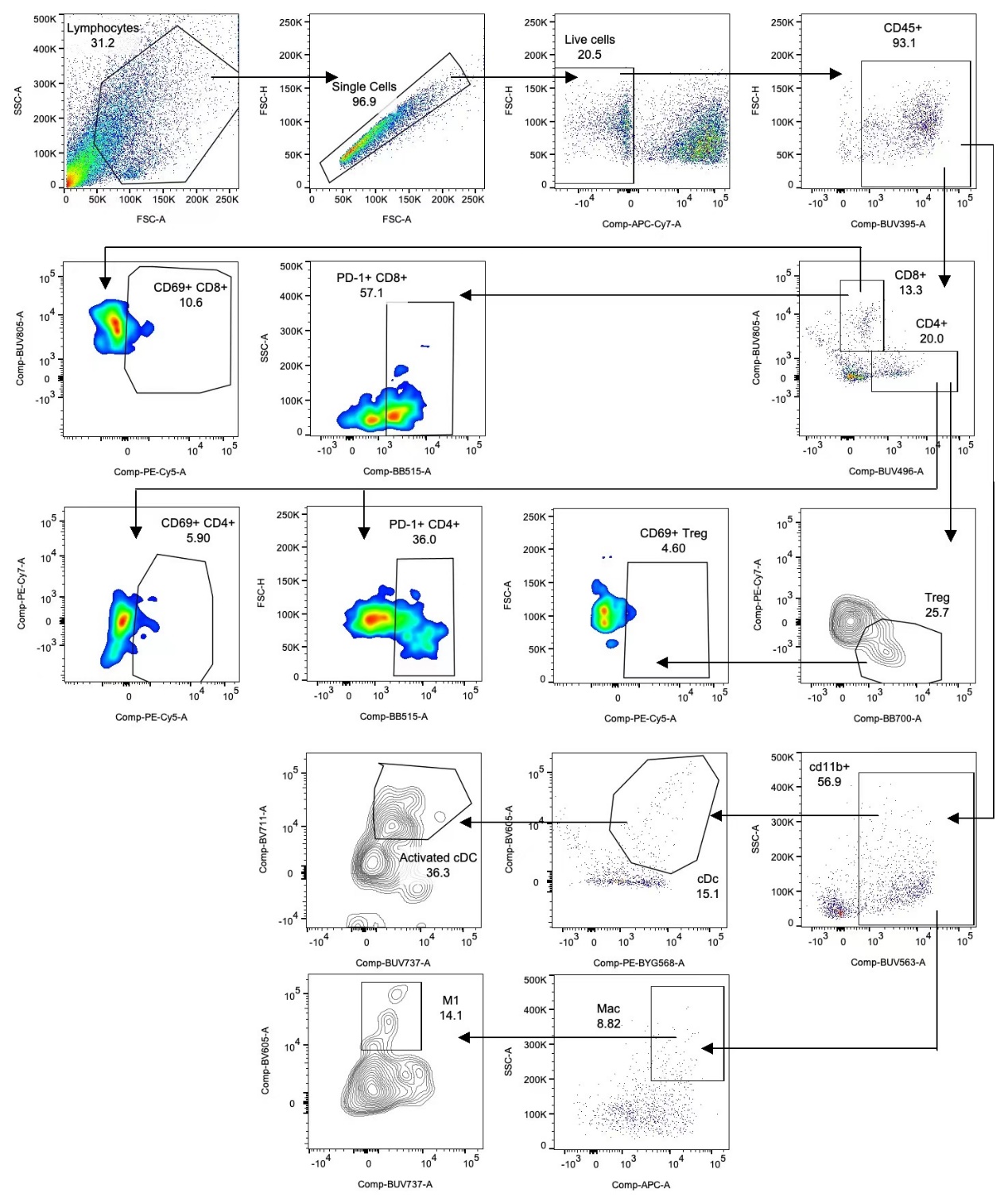


Figure S39. Gating strategy for flow cytometry of cells in tumors.


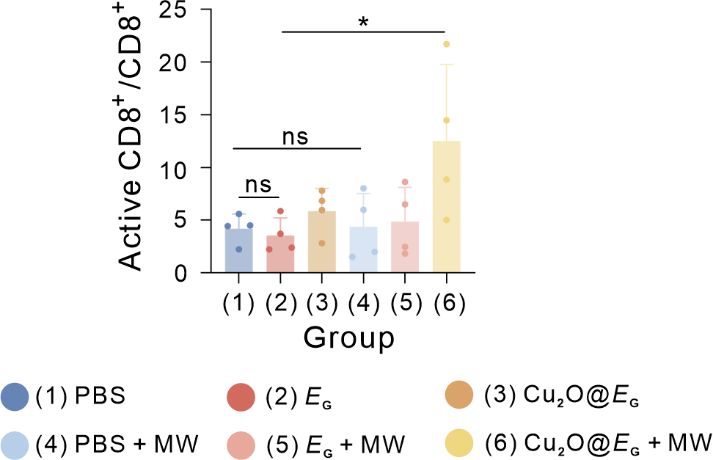


Figure S40. FCM analysis and relative populations of active CD8^+^ T cells in total CD8^+^ T cells upon the indicated treatments. Data are presented as mean ± S.D. (n = 4 mice). Statistical analysis was calculated by using one-way analysis of variance with a Tukey’s test (*P < 0.1 and ns > 0.05).


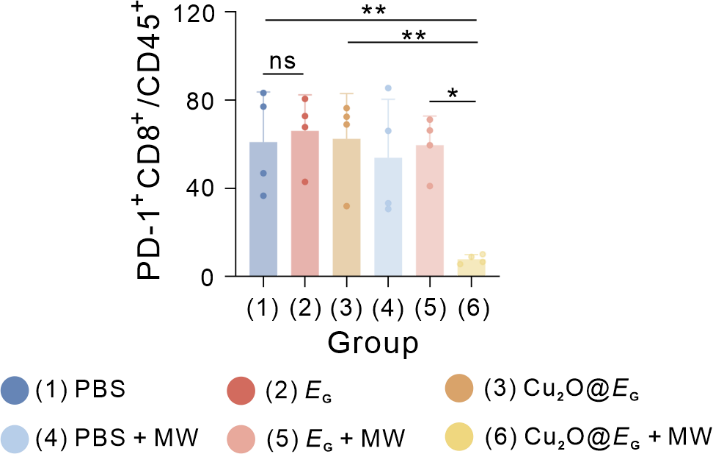


Figure S41. FCM analysis and relative populations of PD-1^+^CD8^+^ T cells in total CD45^+^ T cells upon the indicated treatments. Data are presented as mean ± S.D. (n = 4 mice). Statistical analysis was calculated by using one-way analysis of variance with a Tukey’s test (**P < 0.01, *P < 0.1 and ns > 0.05).
